# Commercial and Scientific Solutions for Blood Glucose Monitoring—A Review

**DOI:** 10.3390/s22020425

**Published:** 2022-01-06

**Authors:** Yirui Xue, Angelika S. Thalmayer, Samuel Zeising, Georg Fischer, Maximilian Lübke

**Affiliations:** Institute for Electronics Engineering, Friedrich-Alexander-Universität Erlangen-Nürnberg, Cauerstr. 9, 91058 Erlangen, Germany; yirui.xue.xue@fau.de (Y.X.); angelika.thalmayer@fau.de (A.S.T.); samuel.zeising@fau.de (S.Z.)

**Keywords:** blood glucose monitoring, Clarke error grid, commercial, diabetes mellitus, machine learning, microwave, non-invasive monitoring, review

## Abstract

Diabetes is a chronic and, according to the state of the art, an incurable disease. Therefore, to treat diabetes, regular blood glucose monitoring is crucial since it is mandatory to mitigate the risk and incidence of hyperglycemia and hypoglycemia. Nowadays, it is common to use blood glucose meters or continuous glucose monitoring via stinging the skin, which is classified as invasive monitoring. In recent decades, non-invasive monitoring has been regarded as a dominant research field. In this paper, electrochemical and electromagnetic non-invasive blood glucose monitoring approaches will be discussed. Thereby, scientific sensor systems are compared to commercial devices by validating the sensor principle and investigating their performance utilizing the Clarke error grid. Additionally, the opportunities to enhance the overall accuracy and stability of non-invasive glucose sensing and even predict blood glucose development to avoid hyperglycemia and hypoglycemia using post-processing and sensor fusion are presented. Overall, the scientific approaches show a comparable accuracy in the Clarke error grid to that of the commercial ones. However, they are in different stages of development and, therefore, need improvement regarding parameter optimization, temperature dependency, or testing with blood under real conditions. Moreover, the size of scientific sensing solutions must be further reduced for a wearable monitoring system.

## 1. Introduction

Diabetes mellitus (DM) is a chronic metabolic disease, which is caused by the lack or ineffective use of insulin produced by the body [1]. According to the report released in December 2020 by the World Health Organisation (WHO), diabetes is among the top 10 causes of death [2]. Overall, patients suffering from diabetes can be categorized in three groups: Type 1 Diabetes Mellitus (T1D), where the body produces no or too little insulin; Type 2 Diabetes Mellitus (T2D), caused by an insulin resistance and Gestational Diabetes Mellitus (TGD) during pregnancy [3].

The normal range of fasting blood glucose is between 70 mg/dL and 100 mg/dL [4,5]. Blood glucose levels (BGL) below 70 mg/dL are called hypoglycemia, whereas levels above 120 mg/dL or 140 mg/dL when fasting or two hours after eating, respectively, or in general a value of >180 mg/dL, correspond to hyperglycemia [6,7]. In case the glucose level differs from the normal range, it can cause an adverse influence on the heart, the blood vessels, the eyes, the kidneys and the nerves [1] as well as circulatory system problems. Those long term complications of hyperglycemia can be categorized into macrovascular diabetic complications, such as heart diseases, and miscrovascular diabetic complications, which cause diseases in organs, such as nephropathy, retinophathy and neuropathy [8]. In particular, the high incidence of nephropathy occurs after suffering diabetes for five-years, and an early indicator thereof is increased urinary albumin excretion [9,10]. The occurrence of retinopathy is caused by the long-term damage to the small blood vessels in the retina, and it is a leading factor of resulting blindness [1,9,11]. Additionally, diabetics have a two- to four-fold higher risk of cardiovascular disease and cardiovascular disease mortality than those without diabetes [3,12,13]. In addition, short term complications of hypoglycemia may lead to coma or death, in the worst cases [14,15,16]. Nevertheless, the complication incidence has declined since the 1990s, benefitting from the better recognition and management of blood glucose levels [13]. Consequently, affected patients must check their blood glucose levels regularly. In 2000, systems for continuous blood glucose monitoring (CGM) became commercially available [17]. These systems automatically measure the blood glucose level and its trend in short time intervals and are, thus, excellent candidates to make the life of a patient suffering from diabetes more comfortable and safe.

From this background, scientists are driven to conduct further research in the field of continuous glucose sensing. For example, in 2014, the biotech-company Verily of Google tried to use a 2-layer smart contact lens combined with a radio chip to monitor blood glucose (BG) variation [18]. However, the results revealed that it is difficult to get a reliable mapping between glucose level in the blood and the tear fluid [19]. In addition, the Robert Bosch company has owned some patents for the BG sensor, which is a sensor that is implantable in the earlobe [19].

**Figure 1:**
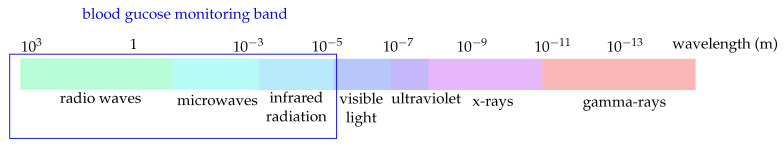
Electromagnetic spectrum.

Various approaches regarding blood glucose sensing have been proposed so far, which can be divided into invasive, minimally invasive and non-invasive as follows:

- Invasive monitoring: The traditional monitoring method of BG is via pricking the fingertip and then putting the obtained drop of blood on the test stripe multiple times per day [19]. This way is called invasive monitoring measurement. Although such monitoring helps patients greatly with BG management and is highly sensitive and correct, it still brings pain, infection risk and even damage to the skin tissue over a long time [20]. Moreover, the finger pricking method falls short when it comes to CGM, since it is conducted every couple of hours by the affected patient rather than in short time intervals over the length of the day [19].
- Minimal invasive monitoring: Minimal invasive glucose sensing is via microneedles inserted into the skin where the interstitial fluid is located [21]. A probe of this liquid is then chemically evaluated to determine the glucose level. The well-established commercial available glucose sensing systems of Dexcom [22] and FreeStyle Libre [23] are based on this method. Herein, a sensor is inserted into the skin of the belly or of the upper arm. After a ‘warm-up’/calibration time of one (FreeStyle Libre 3) or two hours (Dexom G6), the sensor is connected with a wearable device for monitoring the measured data, and the glucose level is determined every few minutes [22,23]. Furthermore, the Dexcom G6 can additionally be calibrated manually by the patient with the BGL measured by finger pricking [22]. Compared to the traditional finger pricking, the determination of the BGL with the FreeStyle Libre or Dexcom sensors is less painful for the patient and yields the significant advantage of enabling CGM. However, the costs for this system are relative high, since the sensor has to be replaced at last every 10 or 14 days.
- Non-invasive (NI) monitoring: NI blood glucose monitoring aims to produce neither pain nor discomfort during the glucose measurement [24]. These approaches can be classified according to the applied glucose sensing method. The primary sensing methods for NI methods are the electrochemical [24,25] and the electromagnetic-based methods [26,27,28]. In electrochemical NI glucose sensors, a probe of saliva [29], tear drop [30] or the exhaled breath [31] is analyzed. The electromagnetic methods are based on the interaction of electromagnetic waves with the human body. The applied wavelengths vary from the m-range (impedance spectroscopy) to the mm-range (microwaves) up to the nm-range (optical frequencies)—compare Figure 1.

Subsequently, post-processing is required to find the relation between the measured signal (usually current, voltage or phase/frequency) and the BG concentration. Indeed, the relation between the measured signal and the blood glucose level (BGL) is often determined by a simple proportionality. However, a calibration step is required to extract the BGL precisely. Nevertheless, since data loss is often a problem, interpolation and extrapolation are also conducted on the data [32]. Furthermore, the rapid development of artificial intelligence (AI) involving machine learning, deep learning and cognitive computing is promising for more accurate and reliable data processing since AI is able to interpret and process high amounts of data [33], and more or less instantaneously, it can suggest a proper recommended course of action to the patient. In sum, this enables further improvements in screening, diagnosis and management of the patients’ diabetes [34]. Methods like a hybrid least squares random sample consensus (LS-RANSAC) [32] or a Principal Component Analysis (PCA) algorithm [35] further enhance the detection sensitivity of the measured sensor data significantly [35].

However, the main focus on using AI in post-processing of BGL data lies in predicting glucose trends [36]. Thereby, the prediction horizon of the BGL is up to 120 min [37], being based on data-based and hybrid models (e.g., Gaussian process and random forest [38]). Various features such as BGL, insulin, meal, exercise, sleep and others can be observed individually and combined with each other to improve prediction accuracy [37]. Additionally, AI-based approaches are also investigated for predicting the risk of secondary diseases [39,40]. However, this is out of the scope of the proposed paper, and detailed information can be found in the review papers [36,37,41,42].

## 2. Non-Invasive Sensor Principles

This article focuses on the non-invasive (NI) sensing of glucose. Consequently, in the following, the main principles of state-of-the-art NI glucose sensing are explored.

### 2.1. Electrochemical NI Sensors

As mentioned in Section 1, NI monitoring can be approached via a medium such as saliva, tear drops or exhaled breath [43]. This is because these body liquids are easily accessible and collected [24]. Principally, a detection of the glucose concentration is also possible via urine, but this is not suitable for a CGM-system [44,45]. Therefore, utilizing saliva, tears and exhaled breath is preferred [46]. In general, such kinds of sensors are called biosensors [25,36].

#### 2.1.1. Saliva Analysis

Saliva contains lots of biological information that reflect the physiology and health status [46]. Thus far it has been widely used in human immunodeficiency virus (HIV) infection diagnosis and drug abuse [47,48,49]. That means saliva indicates physiological functions of the body and can be regarded as an alternative to blood [50]. In the following, the research on biosensors carried out by T. Arakawa et al. in 2016 and their improvement in 2020 will be shown [29,51].

**Figure 2:**
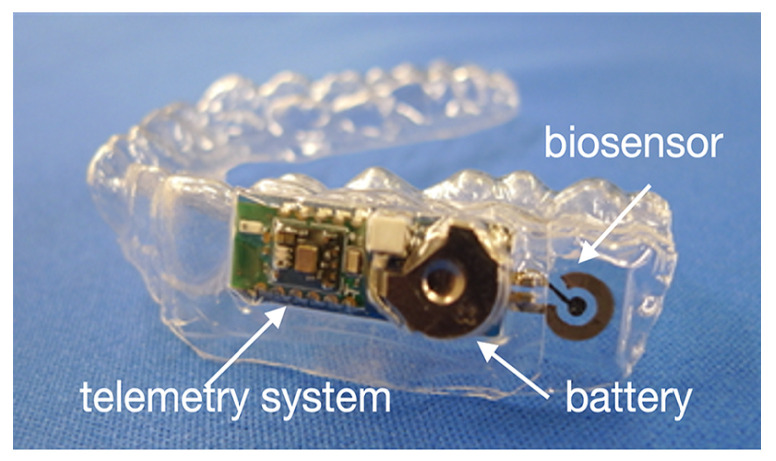
View of saliva-based design (Reprinted with permission from Ref. [29] © 2021 American Chemical Society).

- *Components*: The sensor used for monitoring is called mouthguard (MG) glucose sensor, where an electrode sensor is included. A cellulose acetate (CA) membrane is used as interference rejection membrane to suppress the effect of ascorbic acid (AA) and uric acid (UA), which is the improvement of 2020 [29]. A wireless module integrated with a potentiostat is designed for a continuous measurement. Finally, the results are displayed on a smartphone or tablet device. The design is shown in Figure 2. The first and third layer is in dentition shape and the second layer is for the electrode sensor, wireless instrument and battery.
- *Methods*: The main idea is to use the enzymatic reaction of glucose oxidase (GOD) as the connection between the blood glucose and the saliva. Equations (1) and (2) show the reaction in detail:
  (1) $$\mathrm{Glucose}+O_{2}\overset{GOD}{\to }\mathrm{Gluconolactone}+H_{2}O_{2}$$
  (2) $$H_{2}O_{2}\to 2H^{+}+O_{2}+2e^{-}$$
  The MG glucose sensor in 2020 consists of three layers:
  First layer: It is built with MG material, polyethylene terephthalate glycol (PETG), and modeled in the shape of dentition.
  Second layer: The enzymatic reaction is performed on the electrode sensor, which is formed on the first layer. On the sensor, a GOD mixture is set to the sensing area to detect the glucose concentration. A Bluetooth low energy (BLE)-type wireless measuring instrument is connected with the electrode sensor through a conductive spring with nickel plating for saving the space. The current from the enzymatic reaction will be then transferred via the wireless module and displayed in a smartphone or tablet device via a self-developed application for Android OS. The detection of electrodes shows the correlation to the glucose concentration level.
  Third layer: A second MG material is hermetically mounted through heat welding.
  In 2016, the estimation was realized through artificial saliva, which is composed of salts and various proteins, such as disodium hydrogen phosphate, anhydrous calcium chloride, potassium chloride, sodium chloride, urea and type II mucin from porcine stomachs [51]. Glucose solutions, such as galactose, fructose, mannitol, sorbitol and xylitol, were also prepared for selectivity evaluation. In 2020 further estimations were approached in both artificial and human saliva [29].
- *Results*: The glucose concentration is proportional to the output current. The sensor can detect the glucose concentration in the range of 0–180 mg/dL (1.75–10,000 µmol/L). The saliva glucose concentration of normal and diabetic patients is 0.4–3.6 mg/dL (20–200 µmol/L). The minimum change in glucose concentration can be detected at 0.9 mg/dL (0.05 mmol/L). In addition, experiments were also held to see the performance of the sensor selectivity by comparing the mean relative output current to different glucose solutions with 1.8 mg/dL (100 µmol/L). According to the magnitude of the output current produced by glucose, the glucose is 100%, the glucose solutions like mannitol, sorbitol, fructose and xylitol can be neglected in artificial saliva, and the galactose is only 0.265%. The rejection of AA and UA interference reaches the noise ratio of 97.1%. That means the sensor has a high selectivity. In addition, a stable output can be obtained in approximate 20 min, and the monitoring can last more than 5 h. The glucose concentration of human saliva is obtained after the calibration curve for artificial saliva. In addition, since the sensor works in the oral cavity, the researchers also ensure that the result is stable until four rounds of cleaning, which also indicates that the sensor has excellent waterproofness. For the low glucose level measurement, the designed biosensor has similar accuracy performance with the kit and spectrophotometer with 0.4 mg/dL and 0.3 mg/dL, respectively.
- *Further studies*: Since many other proteins in saliva have influence on the glucose measurement, a sensor, which performs under more complex conditions, is still under research.

#### 2.1.2. Ocular Fluid: Tear

**Figure 3:**
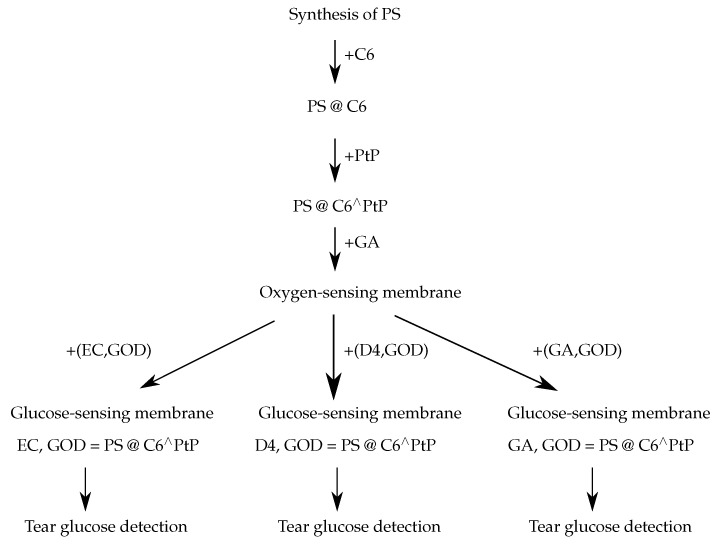
Fabrication of radiometric fluorescent glucose-sensing membranes (Adapted from Ref. [58]).

**Figure 4:**
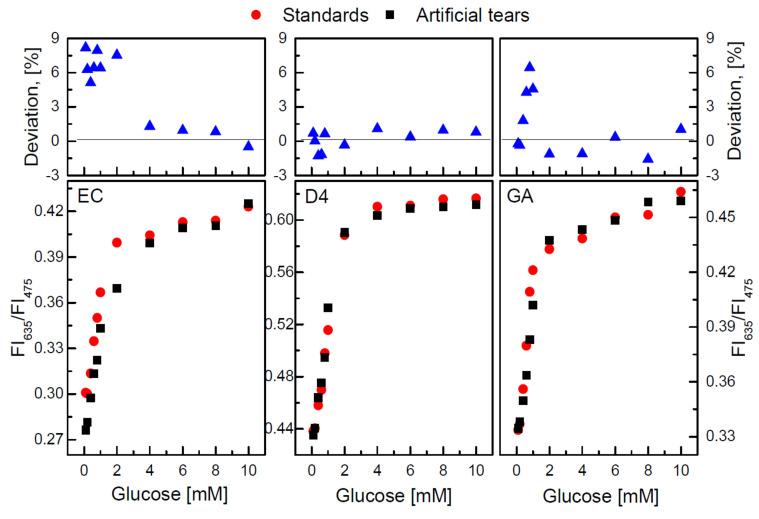
Percentage deviation of radiometric fluorescence intensities and the response between radiometric fluorescence intensities and standard and tear glucose (Reprinted from Ref. [58]).

Tears also carry information and show similar glucose concentration to blood [30,52]. Tears contain organic molecules, which reflect the health status [46]. Compared to saliva, tear glucose concentration is relatively stable in the range of 0.9–90 mg/dL (0.05–5 mM), while blood glucose concentration is in the range of 90–140 mg/dL [53,54]. Therefore, tears have attracted much attraction for decades [55]. In addition, the worldwide use of contact lenses is a strong motivation for tear glucose measurement [56]. In 2014, Verily, which is regarded as the Google Life Sciences, launched the smart contact lens project. Their challenge was to get reliable tear glucose readings [18]. Even though their development was discontinued because the correlation between blood glucose and tear was too weak [18], it is still encouraging to have further innovation based on tear glucose concentration [57]. In the following, a recent research carried out by H.D. Duong et al. [58] is discussed:

- *Components*: The sensor principle is based on the radiometric fluorescent glucose-sensing membranes, which are fabricated based on an oxygen-sensing membrane with different supporting polymers, namely, ethyl cellulose (EC), polyurethanes (such as D4), aminopropyltrimethoxysilane and glycidoxypropyltrimethoxysilane (GA). Figure 3 shows the fabrication of radiometric fluorescent glucose-sensing membranes, which is composed of 2 layers: the oxygen-sensing membrane and GOD on the supporting polymers (EC, D4, GA) [58]. The oxygen-sensing membrane is made of polystyrene particles (PS) doped with oxygen-sensitive fluorescent dye platinum meso-tetra porphyrin (PtP) and Coumarin 6 (C6) (abbreviated with PS@C6⌃PtP) in a sol–gel matrix of GA.
- *Methods*: The main idea is to follow the catalytic reactions. Since the emission band edges of PS@C6⌃PtP appear at 635 nm and 475 nm for PtP and C6, respectively, the ratio of the fluorescence intensities (FI) is chosen at above two emission wavelengths ($FI_{635}$ and $FI_{475}$). The ratio is formed as below, in Equation (3). An excitation wavelength is chosen at 400 nm.
  (3) $$R=FI_{635}/FI_{475}$$
  The investigation is carried out in the artificial tear with the components of 10 mM phosphate saline buffer (PBS, 7.0 pH), 10 µM uric acid, 100 µM ascorbic acid, and 10 µM acetaminophen. To quantify the immobilization properties of GOD of EC, D4 and GA, different amounts GOD are under test, namely, 10, 20, 40, 50, 60 and 100 units (U). The amount of immobilized GOD is in the end 50 U after considering factors such as sensitivity, cost and detection range because too much immobilized enzyme results in a narrow detection range, while too little immobilized enzyme leads to the longer detection time. After deciding the immobilized GOD amount, further measurements of the response, the reversibility, the effect of pH and temperature and the long-term stability on the GOD = PS@C6⌃PtP are estimated. The reversibility of the supporting material indicates the sensitivity. The evaluation thereof is demonstrated by exposing these three materials in a repeated cycle of glucose concentration in the range of 0–36 mg/dL (0–2 mM). Regarding the pH and temperature and since the slope value (SI), the ratio of the FI ratio and glucose concentration, are the vital indicators, the relation between the pH value and the temperature with the slop value should not significantly change. According to this, the 7–9 pH-range and the temperature 30 °C are chosen. The stability is estimated by comparing the FI ratio and corresponding glucose at the beginning and one month later.
- *Results*: The sensor has a detection range of 1.8–180 mg/dL (0.1–10 mM). A high linear sensitivity is given in the low glucose concentration range of 1.8–36 mg/dL (0.1–2 mM). At a high glucose concentration of 36–180 mg/dL (2–10 mM), GA performs better than the other two. Table 1 shows the GOD = PS@C6⌃PtP response to the glucose level of each supporting material in detail.
  For the reversibility, each material performs separately and repeatedly in 0–36 mg/dL (0–2 mM) glucose for about 20 min. Table 2 shows the different relative standard deviation (RSD) of EC, D4 and GA at 0 mg/dL and 36 mg/dL and GA responses faster than EC and D4. For the stability, the FI-glucose curve at the beginning and that from one month later are very close to each other, which means that the membrane has good stability. The SI of the linear calibration curves and the radiometric FI in both standard glucose solution and artificial tears are estimated. Figure 4 gives the final result. Clearly, the SI of both with three different supporting materials has similar performance in standard and tear glucose. The detailed SI values are shown in Table 2. Additionally, the percentage deviation thereof is −1.5 to 9.0%, which also shows a promising detection in the tears.

#### 2.1.3. Exhaled Breath Analysis

Despite of the medium, such as saliva and tear, exhaled breath is another attractive biomarker of diabetes. The relation between diseases and the smell of exhaled breath is well established [31,59]. For instance: ‘fruity smell’ of acetone in the breath can be regarded as an indicator of diabetes, whereas ‘musty and fishy smell’ can be considered as a hint of advanced liver disease [60]. Therefore, exhaled breath analysis provides potential deep insights on physiological and pathophysiological conditions in terms of related diseases [61]. Similar to the other two media, the exhaled breath is, in general, easy to access and collect. In addition, it is safer, friendlier and more acceptable for patients compared to the saliva and tear analysis [31,62]. Examples of biomarkers in human breath are acetone, isopropanol (IPA), carbon monoxide, isoprene and ethanol [31]. The content of acetone is quite large, which can be found both in T1DM and T2DM diabetics, and comes from the increase of acetyl-CoA level in the liver because of the lipolysis [63].

Nevertheless, the correlation between blood glucose level and the detected acetone is controversial as discussed in the literature [64]: positive [65] negative [66,67], some arguing no correlation [68,69,70]. The core problem of BGL-detection based on exhaled breath analysis is that the acetone level is influenced by many factors such as insulin injection, type of diabetes, alcohol intake, exercise, food and beverage intake, etc. [63]. As acetone is produced during fat metabolism, it is also used as a diet marker [71]. Companies, like BIOSENSE, LEVL, Ketonix, Keyto, House of Keto Monitor and KHC M3, have released their breath acetone meters. However, only BIOSENSE, LEVL and Ketonix have received the FDA-approval for diet management and diabetes diagnosis [72]. In the context of diabetes care, however, exhaled breath analysis is currently mainly investigated regarding diabetes diagnosis and not as a CGM sensing system [73,74,75].

#### 2.1.4. Summary

Nowadays research on glucose monitoring based on saliva, tear and exhaled breath is still a hot topic. One of the dominant reasons is that these fluids are easily accessible and can be markers for blood glucose concentration. However, these three MUTs face the same problem: containing various proteins (saliva and tears) or breath biomarkers (exhaled breath), which raises the challenge of having accurate monitoring. This also indicates that in future studies more interference rejection parts are needed for a better monitoring. Moreover, the lag time between blood and tear glucose, and exhaled breath is different. The lag time of blood and tear glucose is about 15 min, whereas the lag time of exhaled breath depends on the type of the sensor [76]. Therefore, reducing the lag time during the secondary fluid glucose monitoring is another important research point as well [77,78,79]. Furthermore, since saliva and tears are human parts, the materials also need to be bio-materials, which brings the issue of allergies, such as skin irritation and rejection reaction [80].

### 2.2. Electromagnetic Non-Invasive Monitoring

Optical techniques utilize the reflection, absorption and scattering properties of waves. Well-known methods are for example Raman spectroscopy, optical polarimetry (OP) or optical coherence tomography (OCT) [77]. The millimeter and microwave sensing and bio-impedance spectroscopy utilize the dielectric properties of glucose [28,77]. Both techniques are applied mostly over the skin. However, the tissue surface is rough, which is one main factor leading to scattering and energy loss. Such characteristics of tissues lead to another vital point, the so-called penetration depth. If the penetration depth is not high enough, it is hardly possible to reach the vessels, i.e., arteries in the body for sensing the glucose change. In consequence, the monitoring accuracy will be reduced [77]. Equation (4) shows the calculation of the penetration depth $D_{p}$, where $f_{r}$ is the resonating frequency, *c* represents the electric wave speed in free space, $\varepsilon_{\mathrm{sam}}$ stands for the dielectric constant, and tanδ represents the loss factor of the deposited sample on the sensing area. Normally the skin thickness is over 1 mm, which means the penetration depth should be greater than this value. In this section, Raman spectroscopy and microwave-based monitoring methods will be explored in detail, especially the microwave-based ones.

(4) $$D_{p}=\frac{c}{2\cdot \pi \cdot f_{r}\cdot {\left(2\varepsilon_{\mathrm{sam}}\right)}^{0.5}}{\left[{\left(1+{\left(\tan\delta \right)}^{2}\right)}^{0.5}-1\right]}^{-0.5}$$

#### 2.2.1. Raman Spectroscopy

**Figure 5:**
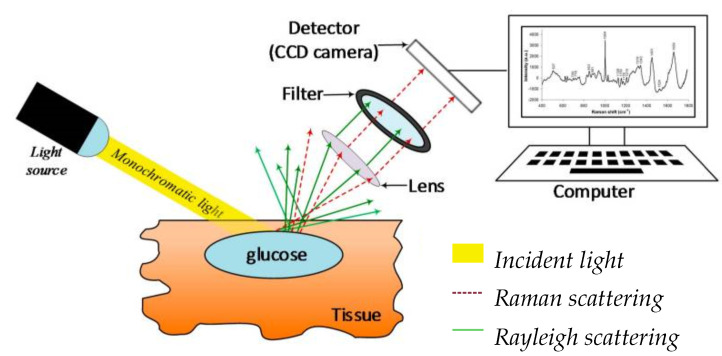
Scheme of a applied BGL measurement setup using Raman spectroscopy (Adapted from Ref. [77]).

It is a vibrational spectroscopic technique based on Raman scattering. The rotational and vibrational states among molecules are the dominant factors in Raman spectroscopy, resulting in the so-called Raman peak in the spectrum. Another important feature is the Raman shift (with the unit ${\mathrm{cm}}^{-1}$), which is the difference between the initial and vibrational wavelengths. A basic Raman spectroscopy consists of 4 parts, namely a monochromatic light source, a lens, a filter and a detector connected to the computer. Figure 5 gives an overview of basic Raman spectroscopy. The reason why Raman spectroscopy is preferred is that it has high sensitivity to detect tiny changes with a molecular size of 1 µm [81,82]. The general advantages of Raman spectroscopy are higher depth penetration compared to mid-infrared spectroscopy, being less sensitive to temperature changes compared to OCT, wide application and high specificity [77]. In the following, recent studies by J. W. Kang et al. [83] and Y. S. Park et al. [84] are discussed.

J. W. Kang et al. aim to show that in vivo glucose measurement can be achieved by a direct observation of Raman peak [83].

**Figure 6:**
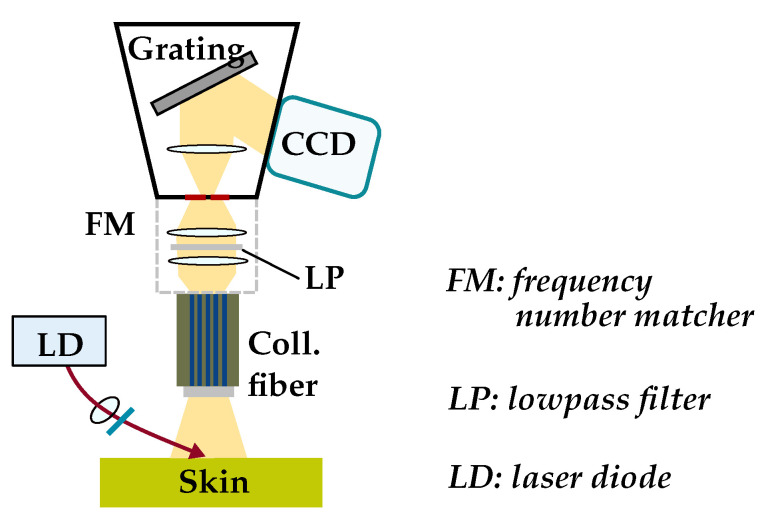
Scheme of Raman spectroscopy system (Adapted from Ref. [83]).
- *Methods*: The measurements were conducted on three female Yorkshire pig ears. In the experiments, four glucose difference solutions (ΔG) of 456 mg/dL, 371 mg/dL, 352 mg/dL and 256 mg/dL are used to find the relation between the intensity of increase of the Raman peaks and the glucose difference. A high linearity is found between the Raman peak intensity, the band-area ratio and the glucose difference, respectively. The band-area ratio is the normalized glucose intensity between the glucose Raman peak intensity and the dominant tissue Raman peak intensity. To maximize the effective sampling volume and for stability reasons, an off-axis Raman instrument (Figure 7) is designed with a configuration of an oblique angle of 60° laser illumination. For temperature controlling a water blanket is used. The penetration depth is beyond 1 mm under the skin.
- *Results*: The detectable glucose concentration is between 29 and 78 mg/dL. The tests are arranged for two five-minute measurements or 10 min in total for spectra collection. It was highlighted that the oblique angle incidence of the laser proved a more effective way to measure the glucose Raman signal. Nevertheless, there are challenges such as movement of the object, sweat, temperature, heart rate, a lower maximum glucose concentration, less integration time, smaller system size and adaption to more users physical situations from different countries. In further development, improving the Raman system and developing sophisticated prediction algorithms are the main directions of J.W. Kang et al. [83].

- *Components*: The design consists of the following parts: An 830 nm laser diode, an imaging spectroscope that includes a mechanical shutter, a charge-coupled device (CCD sensor) and a filtered laser beam of 250 mW with an incidence angle of 60${}^{\circ }$. In Figure 6 the scheme of the Raman spectroscopy system is depicted.

**Figure 7:**
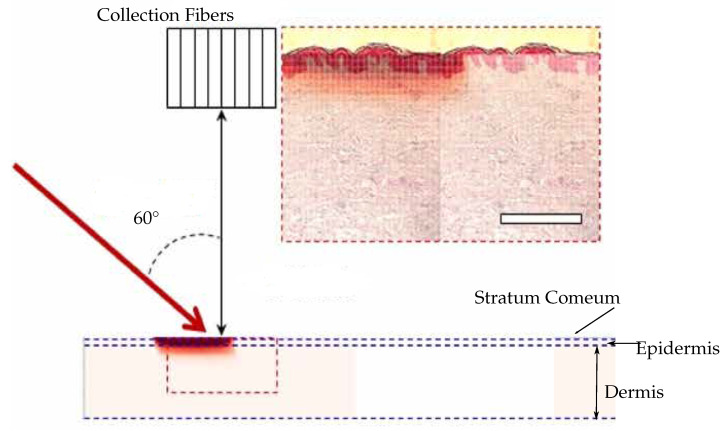
The off-axis Raman instrument (Adapted from Ref. [83]).

Y.S. Park et al. [84] have conducted an additional study to J.W. Kang et al. As mentioned before, the miniaturization of the system needed further improvement. Y.S. Park et al. identified the trade-off between the Raman spectrometer collection efficiency and the device miniaturization. The setup of the glucose concentration was in the range of 50 to 400 mg/dL with uniform distribution.

- *Methods*: The framework of this research is as follows: Raman spectra serve as the input in the computer space. The spectral information containing the glucose information is then processed inversely through a back regression analysis, so that the prediction performance can be evaluated. Random forest (RF) regression and partial least squares regression (PLSR) are utilized for the prediction. A Monte-Carlo simulation is used to demonstrate the Raman photon generation in skin tissue, which consists of excitation photons scattered into the skin tissue and Raman photons escaping from the skin surface. The excitation laser transmits at 785 nm with 60 mW. The glucose concentration is set to be in the range of 50–400 mg/dL, and the collection efficiency is set five times halved from 3.2% to 0.2%.
  Equations (5)–(7) show the all used spectra in this research in more detail. The computer simulates a glucose spectrum $S_{\mathrm{glu}}\left(\lambda ,g,t\right)$, a fluorescence background spectrum $S_{\mathrm{fluo}}\left(\lambda ,t\right)$ and a total spectrum $S_{\mathrm{total}}\left(\lambda ,g,t\right)$, which is the sum of the former two spectra at a certain glucose concentration. In addition, the simulation of excitation photons and Raman photons is also included, which is introduced for the efficiency calculation. The parameters λ, *g*, *t* correspond to the wavelength, glucose concentration and elapsed time, respectively. The glucose spectrum is calculated by the glucose spectrum simulated by the Monte-Carlo method at a given concentration $S_{\mathrm{glu}\_\mathrm{scaled}}\left(\lambda \right)$, considering the Raman scattering $\eta_{R}\left(g,t\right)$, the collection efficiency $\eta_{\mathrm{collect}}$ indicating the miniaturization, the excitation laser power $P_{\mathrm{ex}}$, the excitation photons $\lambda_{\mathrm{ex}}$, the Planck constant *h* and the speed of light *c*. The fluorescence background spectrum is measured for a duration of 30 s with an excitation laser and a lensed fiber optic probe. In addition, α is the time-decay constant.
  (5) $$\begin{array}{cc} S_{\mathrm{total}}\left(\lambda ,g,t\right) & =S_{\mathrm{glu}}\left(\lambda ,g,t\right)+S_{\mathrm{fluo}}\left(\lambda ,t\right) \end{array}$$
  (6) $$\;\;\;\;\;\;\;\;\;\;\;\;\;\;\;\;\;\;\;\;\;\;\;\;\;\;\;\;S_{\mathrm{glu}}\left(\lambda ,g,t\right)=S_{\mathrm{glu}\_\mathrm{scaled}}\left(\lambda \right)\cdot \eta_{R}\left(g,t\right)\cdot \eta_{\mathrm{collect}}\cdot \left(P_{\mathrm{ex}}\cdot \frac{\lambda_{\mathrm{ex}}}{h\cdot c}\right)$$
  (7) $$\;\;\;\;\;\;\;\;\;\;S_{\mathrm{fluo}}\left(\lambda ,t\right)=S_{\mathrm{fluo}\_\mathrm{measured}}\left(\lambda \right)\cdot \exp\left(-\alpha t\right)$$
- *Results*: A wavelength of 1125 ${\mathrm{cm}}^{-1}$ is mostly used for glucose detection. However, it is still a challenge for the non-invasive glucose monitoring devices to have a good trade-off between the performance and the miniaturization with respect to the collection efficiency. Here, three criteria are used to evaluate the prediction, namely, the regression analysis (R), the mean absolute relative difference (MARD), and the zones A and B in the Clarke error grid (CEG A + B). With the reduced collection efficiency (from 3.2% to 0.2%), the achieved results get worse, as depicted in Table 3. For further studies, artifacts detecting subject movement and sweating could be considered as improvement aspects.

#### 2.2.2. Impedance Spectroscopy-Based Monitoring

For more than 15 years, impedance spectroscopy or also dielectric spectroscopy has been under research for non-invasive glucose sensing [85]. The research of numerous scientists resulted in a CE approval for such an impedance spectroscopy-based sensor called Pendra in 2003. However, post-marketing studies on six type 1 diabetes patients revealed that 4.3% of the Pendra readings were in the dangerous Zone E of the Clarke error grid. Consequently, Pendra was removed from the market shortly after its CE approval [86]. Moreover, GlucoBand is another impedance spectroscopy-based glucose monitoring system with a similar fate as Pendra and was never released in the commercial market.

In impedance spectroscopy, the impedance *Z* of human tissue is measured by passing alternating current signals across the skin in the frequency spectrum of 100 Hz to 200 MHz [85,87,88]. The specific reaction of blood and tissue cells to a change in glucose level results in a change in the electrolyte balance across the membranes of blood and underlying tissue. Therefore, the electric conductivity σ, and thus, *Z* of tissue is sensitive to the glucose level [85]. However, non-invasive glucose sensing with impedance spectroscopy is challenging due to distortions imposed by the movement of the electrodes on the skin surface [89], sweat, and temperature fluctuations as well as skin thickness or moisture variations [88]. Therefore, researchers proposed to combine impedance spectroscopy sensors with multiple sensors to increase the overall accuracy and stability of non-invasive glucose sensing. In [87], a wearable system comprising impedance spectroscopy, temperature, humidity as well as optical sensors. Moreover, in [89], a similar multisensor wearable system was proposed. The system consisted of dielectric, optical, temperature, humidity sensors and an accelerometer. Both approaches fused several physiological parameters to increase the overall sensor accuracy.

**Figure 8:**
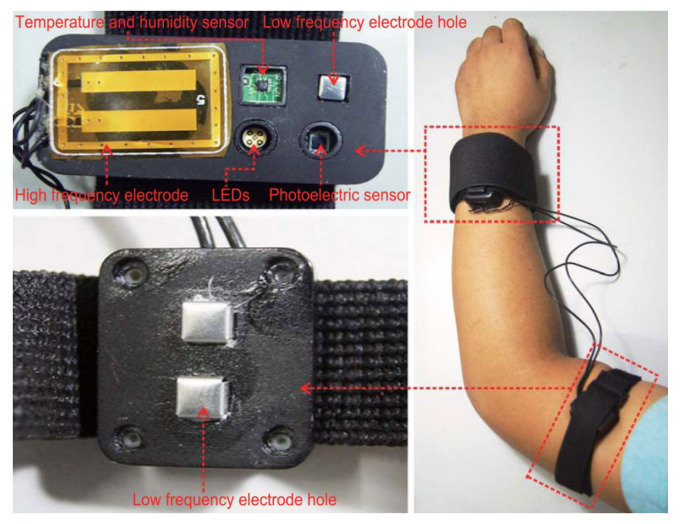
Wearable multisensor platform for non-invasive glucose sensing. The system includes impedance spectroscopy-based sensors (electrodes) as well as optical sensors (LEDs, photoelectric sensor) and humidity and temperature sensors (Reprinted from Ref. [87]).
- *Methods*: The described setup for non-invasive continuous glucose monitoring was evaluated in 33 experiments on six healthy subjects and three subjects with diabetes. The system comprised two different electrodes for impedance spectroscopy. The signal frequency applied on the low-frequency electrodes on the waist and upper arm was 1 kHz to 150 kHz and on the high-frequency electrodes on the waist 10 MHz to 60 MHz, respectively. Therefore, the low-frequency electrodes measured the impedance of the arm and the high-frequency electrodes the impedance of the waist tissue. The use of multiple sensors and the application of time series analysis on these signals endured the time delay between the physiological parameters and the glucose level change. The performance of the system was evaluated by comparing the estimated glucose profiles with the reference glucose profiles derived by finger pricking or a dynamic glucometer.
- *Results*: The results indicated that the average of the correlation coefficients of the estimated and reference glucose profiles was 0.8315. Moreover, the normalized root mean squared error (NRMSE) was 14.6064. Overall, 100% of the estimated glucose levels fell in Zones A and B of the Clark error grid, and 92.86% fell in Zone A. Therefore, it was concluded that the proposed system had the potential of accurate non-invasive continuous glucose monitoring.
- *Limitations:* The work of Geng et al. is promising; however, the movement of artifacts due to relative displacement between the electrodes and the skin was not addressed. In future research, this problem could be explored.

Research by Geng et al. (2017) [87]:

- *Components*: The wearable system for non-invasive glucose motioning proposed in [87] consisted of multiple sensors (Figure 8). A flexible band worn on the wrist contained temperature and humidity sensors as well as electrodes for impedance spectroscopy. Moreover, LEDs and a photoelectric sensor were integrated into the flexible band for optical sensing. Additionally, a flexible band with an electrode for impedance spectroscopy was fixed to the upper arm.

#### 2.2.3. Microwave-Based Monitoring

Reviewing the microwave-based system, utilizing frequencies in the GHz-range, is one of the main goals of the proposed work and is meant to have a great potential in biosensors. This is because of its promising characteristics: high penetration depth in human tissue, high sensitivity to subtle variation of glucose concentration and easy and low-cost fabrication as well as for safety reasons [77,90,91]. In general, the technology can be classified into three parts according to their properties, namely, reflection, transmission and resonant perturbation [77]. The reflection-based technique is a one-port one, which evaluates the reflection parameter $S_{11}$, as there is a dependency relation between the intensity and phase variation of the signal and permittivity variation in the blood glucose level [77]. The transmission-based technique on the other hand is a two-port technique, which utilizes the transmission coefficients $S_{21}$ and reflection coefficients $S_{11}$, whereas the resonant perturbation-based technique uses the Q-factor [53,77]. In the following, two early findings and several recent studies are described.

Early Findings

In this subsection, two early findings will be introduced, which are both given by M. Hofmann et al. [92,93].

- In 2011, an approach based on the dielectric and mounting properties of human tissues was demonstrated [92]. According to it, different components can be composed to simulate a tissue model. A model with skin, fat and blood vessels was proposed to mimic human tissues. The glucose detection range is 50–500 mg/dL. Two different types of patch antennas are applied to find the relation between the scattering parameters and the blood glucose concentration. The simulation is based on the crook of the arm model. The human arm model is represented via the previously published permittivity [94]. The simulation uses $S_{11}$ and $S_{21}$ in the range of 5 to 12 GHz.
  The setup was tested for a water plus glucose test fluid, as water has a high permittivity but a similar dielectric behavior to blood. The measuring frequency is 5–6 GHz. The tested water–glucose solution is from 50 mg/dL to 500 mg/dL, and a slope of 392.5 kHz/dL is achieved between 5.3 GHz and 5.5 GHz.
- In 2013, a six-port reflectometer and a homodyne vector network analyzer were used, with basis on the dielectric measurements as well [93]. In this study, the glucose detection range is 72–500 mg/dL. Both reflection and transmission methods are used and indicate that there should be a limitation of maximum propagation distance in the material under test (MUT) and a proper choice of the waveguide parameters. In addition, a simplification model and an effective permittivity were introduced. The bold line with width *w* represents the conductor and the gray part is the MUT. Additionally, *h* is the substrate thickness of 100 µm, ω corresponds to the angular frequency, and χ serves as the glucose concentration. The model is simplified from a waveguide cross section of a stacked structure with a microstrip line covered by a blood vessel. As the radius of the blood vessel is larger than the conductor width, the blood vessel is regarded as the MUT, so that the transmission along the blood vessel can be achieved. It has to be remarked that there is no air gap between the microstrip line (MSL) and MUT.
  In the experiment, 40 samples of real blood with added NaCl and water are used, where NaCl and water serve as the carrier and reference samples. A total of 50 measurements on the MUT were conducted with two 2.92 mm connectors in 14–16 GHz with a glucose concentration variation between 0 and 40.000 mg/dL. The output magnitude and phase shift of both reflected signal and transmitted signal are summarized in Table 4. This implies that when the glucose concentration varies from 0 mg/dL to 10.000 mg/dL, a phase shift of 0.08${}^{\circ }$ and a transmitted magnitude change of 3.2 mV occurred for the reflected signal, whereas for the transmitted signal a phase shift of 0.2${}^{\circ }$ and a transmitted magnitude change of 8 mV were shown. Such a variation on the transmitted magnitude and phase shows the sensitivity of the proposed sensor. That means both reflection-based and transmission-based sensors can detect the minor glucose concentration variation.

**Table 4:** Output magnitude and phase shift of reflected signal and transmission signal according to the results of [93].

	Reflected Signal		Transmission Signal	
	Magnitude (mV)	Phase Shift (°)	Magnitude (mV)	Phase Shift (°)
Δχ = 0 mg/dL	∼894.4	∼−3.8	∼858	—
Δχ = 10.000 mg/dL	∼891.2	∼−3.96	∼864	∼−7.76

Recent Findings 

In the following four recent new studies are introduced.

Research by A.E. Omer et al. [35,95]:

The remarkable points of this research are that the sensor is portable and the raw data from the radar receiving channel demonstrate a clear correlation for a change in the blood glucose level. The first findings were published in [95] which was extended in [35].

**Figure 9:**
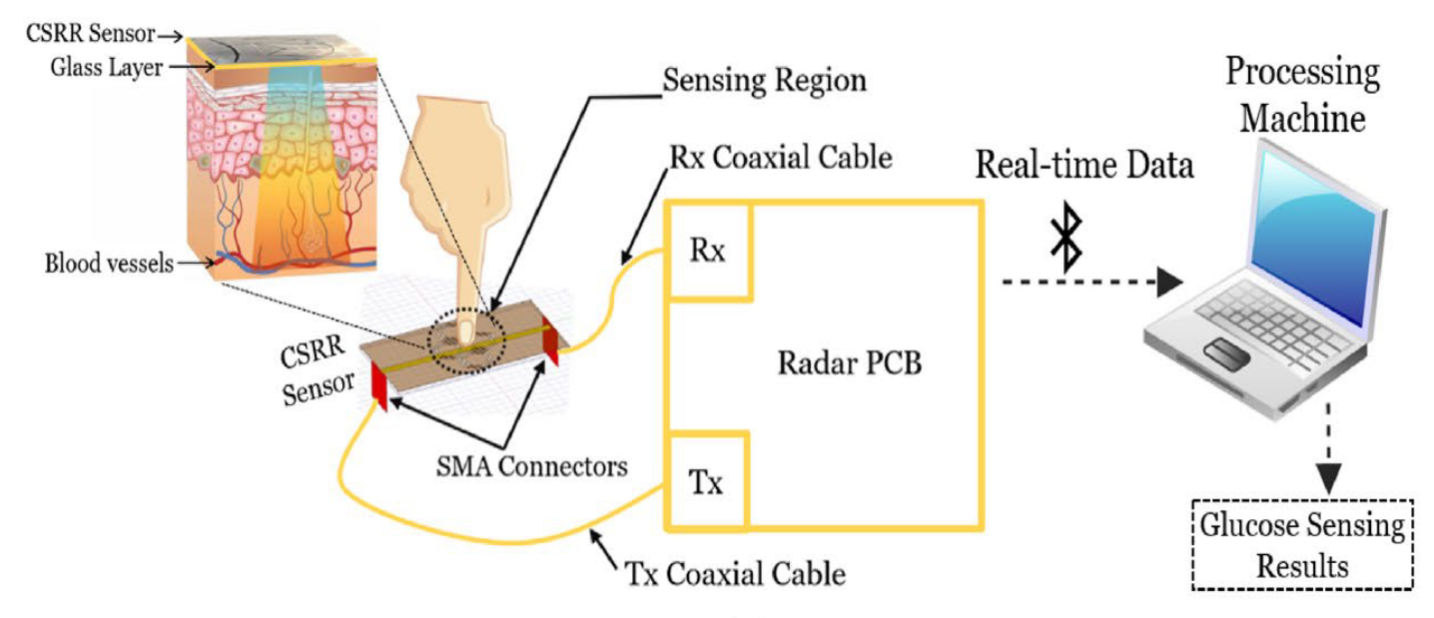
Working principle of the proposed sensor system by Omer et al. The portable radar-driven sensor measures the BGL by sensing electromagnetic waves of small wavelengths into the blood vessels of the fingertip (Reprinted from Ref. [35]).
- *Results*: From the numerical analysis, a clear frequency shift occurs after placing a container on the sensor. After loading with distilled water, a further frequency shift and a steeper magnitude variation on the $S_{21}$ can be observed. Additionally, a clear separable change on both frequency and amplitude can be directly seen after different glucose samples in the range of 40–500 mg/dL are filled into the container. Notably, the skin thickness has a negative influence on the coupled electric field, implying the amplitude variation per dielectric permittivity and the loss tangent are different. In the in vitro VNA measurements, both sample concentration ranges show similar responses in the frequency shift and amplitude variation for the two typologies. Those changes can be related correspondingly to the different sample concentrations. Remarkably, only a slight variation occurs in the frequency shift, whereas the amplitude variation is significant. Nevertheless, the average monitoring sensitivity is 0.94 MHz/(mg/dL) (dispersed: 0.45–0.95 MHz/(mg/dL); compact: 0.63–1.25 MHz/(mg/dL)), which indicates that the determined variation can be as little as 1 mg/dL. Additionally, after applying PCA post-processing, a clearer separation can be seen for different sample concentrations of 70, 90 and 110 mg/dL. In the in vivo test, the designed sensor operates together with a radar system on a 29-year old healthy male volunteer for a maximal test duration about one minute. The test result indicates the same glucose variation trend as with a glucometer. Moreover, the result curves are distinct and separable for different glucose concentrations.

- *Components*: The idea of the sensor design is based on the hexagonal-shaped complementary split ring resonator (CSRR). The proposed sensor is composed of four-cell CSRRs in a honey-cell pattern with two different topologies, namely, a compact one with a horizontal distance of 7.6 mm and a dispersed one with a horizontal distance of 12.6 mm. The distance between the other two vertical placed CSRRs is 12 mm. Additionally, a VNA, a 2.45 GHz radar board and a container are used in the setup.
- *Methods*: The experimented glucose level varies in the range of 70–120 mg/dL on the blood mimicking aqueous solutions. The chosen operation frequency is 2.45 GHz, as it not only matches the Industrial, Scientific and Medical (ISM) band for the sensor integrating in the radar system but also provides adequate penetration depth for the glucose detection. As the magnitude and phase variation is more distinct and sensitive on the transmission coefficient $S_{21}$ than on the reflection coefficient $S_{11}$, the sensitivity evaluation is only conducted through frequency shifts of $S_{21}$. The whole research can be divided into three parts: a numerical analysis, an in vitro VNA measurement and an in vivo evaluation with a radar system. The numerical analysis can be further divided into three cases: unloaded, loaded with an empty container and loaded with pure distilled water of volume 600 µL on the compact and dispersed CSRR sensor. Additionally, different skin layer thicknesses of 0.5, 1.0 and 1.5 mm are considered in the simulation. In the in vitro VNA measurement, glucose concentrations of 70–120 mg/dL and 200–500 mg/dL are applied to the two different sensor topologies. For the sake of sensitivity improvement, a PCA algorithm is utilized to analyze the scattering response of the VNA measurement results. Finally, the in vivo test is investigated on the finger. Different to the measurement experiment (depicted in Figure 9), the costly and bulky VNA is replaced with a radar system, which is low cost and low power and carries out real-time monitoring. Notably, the whole research is carried out at room temperature or 25 ± 1 ℃.

Research by V.V. Deshmukh et al. in 2020 [96]:

Microstrip antennas have various structures, which lead to different antenna characteristics. In 2020, V.V. Deshmukh et al. [96] gave suggestions for the choice of antenna structure used in NI-measurements. The compared antennas are rectangular spiral shaped antennas, ultra wide band (UWB) antennas and narrow band antennas. In 2021, V.V. Deshmukh et al. [97] extended their findings by using a narrow band microstrip antenna to measure the BG level, which will be shown later in the subsequent section.

- *Methods*: The experiments were conducted in the range of 1 GHz to 5 GHz. Blood glucose levels between 0 and 400 mg/dL were measured via a VNA. The antenna is fabricated on FR4 material with 1.6 mm height and a single feed line with a 50 Ω characteristic impedance. The spiral antenna, UWB antenna and narrow band antenna are designed for resonating frequencies of 4.7 GHz, 3.4 GHz and 1 GHz, respectively. The relation to the BGL is constructed according to the return loss measured by $S_{11}$ and the frequency peak.
- *Results*: Because of the rectangular spiral shape of the antenna, there is a certain loss, characterized by the return loss of $S_{11}$, between simulated and measured values. The simulated value and the tested values of the $S_{11}$ peak for the spiral shaped antenna are –32.04 dB and –20.09 dB, respectively. As the frequency response of UWB antenna is quite wide, it is not easy to get a correspondence of the frequency response and the blood glucose level, although it is an encouraging method. The final simulated and tested values of the $S_{11}$ peak are –33.327 dB and –29.236 dB, and –42.22 dB and –39.34 dB, respectively. Nevertheless, the narrow band antenna gives a more accurate linear response compared to the other two antennas. For the narrow band antenna, the simulated value and tested value of the peak of $S_{11}$ are –31.37 dB and –26.84 dB, respectively. In sum, the loss for the narrow band antenna is the smallest, compared to the other two types of antennas.
- *Further Improvements*: As next steps, V.V. Deshmukh et al. [96] suggest to generate a mathematical model to predict blood glucose level. For this purpose, the forehand data can be collected to build a data set.

Research by V.V. Deshmukh et al. in 2021 [97]:

- *Methods*: The main idea of this research is based on the observed frequency shift of $S_{11}$ depending on the variation of BGL. The designed narrow band microstrip antenna resonates at 1.36 GHz. The whole research has two parts: data collection and data processing. The BGL is in the range of 0–400 mg/dL.
  In the data collection, 250 individuals were involved: 75 diabetic subjects (50 male and 25 female aged 18–65), 50 pre-diabetic subjects (25 male and 25 female aged 18–65) and 125 diabetic subjects (75 male and 50 female aged 10–70). The Accu-Check machine was used to record the reference BGL and a VNA to collect corresponding frequency shifts. The reference BGL data were collected 12 h after the fasting period or 2 h after the lunch.
  In the data processing part, some redundant data were eliminated, which resulted from the environmental change interference. After that, two regression analyses were performed, linear regression with and without sub band frequency analyses, which depended on the frequency shifts. The detailed 3 sub bands are the frequency shifts less than 1 GHz (non diabetic), 1–1.5 GHz (pre-diabetic) and larger than 1.15 GHz (diabetic).
- *Results*: The performance without the sub band results in the coefficient of determination value $R^{2}$ of 0.7525, the surveillance error grid (SEG), which is an estimation for clinical scenarios. SEG illustrates the risk level through a color-coded graph from dark green to dark red. Lower risk is displayed in dark green, whereas higher risk is shown in dark red. In other words, the result with more points appears in the green part and the one with less points in the red part performs better. The SEG results are 60.91% in the dark green part, 20% in the green part and 19.09% in the yellow part, and the MARD result is 22.98%. After using the sub-band regression, the performance is improved: $R^{2}$ of 0.8479 for non-diabetic, of 0.8346 for pre-diabetic and 0.9133 for the person with diabetes; SEG of 85.37%, 9.76% and 4.88% in the dark-green, green and yellow parts, respectively, and mean MARD of 4.204%.
- *Further Improvements*: The observed measurement errors, such as in the skin thickness and the finger pattern, are mainly caused by the finger movement, the temperature, the pressure and the humidity. Therefore, a proper analysis is important for the microwave-based BG sensor. Meanwhile, the finger should be pressed moderately on the sensor, otherwise the blood may be pressed away, which may lead to measurement inaccuracy.

Research by X. Xiao et al. [32]:

Although it is quite difficult to find the correlation between the frequency response and the actual glucose concentration by using UWB microwave, X. Xiao et al. proposed a novel method, the so called UWB microwave with improved neural network and hybrid optimization (INNHO). Both S-parameter and frequency response are utilized. The results are remarkable, as the prediction error is just in the range from 0.31% to 4.64% for a glucose concentration in the range of 0–500 mg/dL. The testing object is a glucose–water solution.

- *Methods*: The whole process can be divided into two parts, namely, a detection part and a data processing part. In the detection part, a VNA and a three-layer earlobe model are employed, which play a vital role in further data processing and serve as training dataset and test set. The VNA is calibrated with a 12-term short-open-load-thru (SOLT), including isolation using OLSN50 calibration kits. The VNA is operated in the range of 0.2–4 GHz and with a stepsize of 6.25 MHz to detect the reflection and transmission coefficients. In the earlobe model, B1 corresponds to the blood layer with a thickness of 3 mm, whereas F1 and F2 are two fat layers with the thickness of 1.5 mm each. D1 and D2 are the boundaries between the fat and blood layer. A1 and A2 represent two antennas with a size of 80 mm × 20 mm, whereas E describes the plane Electromagnetic (EM) wave. In particular, $E_{1i}$, $E_{1r}$, $E_{2i}$, $E_{2r}$ and $E_{3}$ are the transmitted wave in layer F1, the reflected wave in layer F1, the transmitted wave in layer B1, the reflected wave in layer B1 and the transmitted wave in layer F2, respectively. Additionally, $\varepsilon_{i}$ and $\sigma_{i}$ are the permittivity and conductivity of the respective medium. The measured data are then stored in a PC for further signal post-processing.
  In the subsequent processing part, the INNHO is used, which consists of two models: a modified back propagation neural network (BPNN) model and a hybrid least squares-random sample consensus (LS-RANSAC) model. Since this research uses machine learning signal post-processing, the detailed methods will be explained in Section 3.1.
- *Further Improvements*: The model in this state misses real-world factors to some extent. Thus, e.g., the thickness of the tissue should be considered.

**Figure 10:**
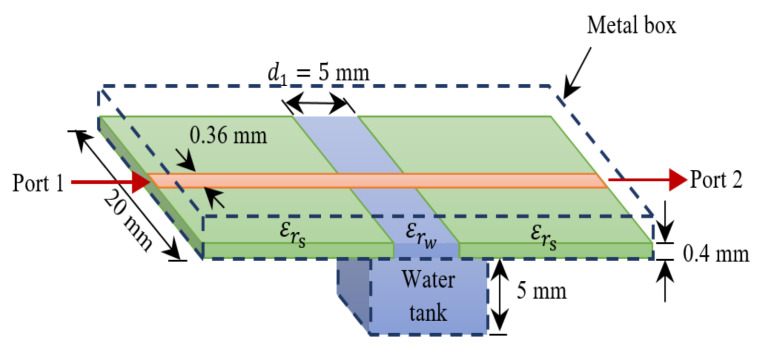
View of the proposed sensor approach (Reprinted with permission from Ref. [98] *©* 2020 IEEE).
- *Methods:* The main idea is to evaluate the frequency shift of the reflection coefficient $S_{11}$ caused by various glucose concentrations. Moreover, two step-sizes are chosen for studying the relation between the glucose concentration and the reflection and the transmission coefficients, a fine one with 10 mg/dL in the range of 0–110 mg/dL and a coarse one with 50 mg/dL in the range of 0–500 mg/dL.
- *Results:* The corresponding phase variation to the glucose concentration change is detected through the reflection coefficients $S_{11}$, with circa 2 ° per 10 mg/dL change and with about 10 ° per 50 mg/dL variation, while no significant phase difference, with 0.035 ° per 10 mg/dL variation, can be seen through the transmission coefficients $S_{21}$ (Figure 11), which indicates that the phase variation of $S_{11}$ is more sensitive than of $S_{21}$.
- *Challenging:* The fabrication of the sensor is challenging because of the high matching purpose, which means an impedance-tuner is needed to avoid the mismatching. Additionally, a cable with a high phase stability is in demand. Furthermore, the material of the substrate and an additional layer above it need to be moisture-absorption free.

Research by S. Zeising et al. [98]:

- *Components:* The approached sensor (Figure 10) is a two-port microstripline simulation based system with an operating frequency of 19.037 GHz after tapering and an impedance of 50 Ω. It is adhered to a substrate with a dielectric constant $\varepsilon_{r_{s}}$ of 10.7. The size of the sensor is 0.36 mm in width. The MUT object is a water glucose solution of 0–500 mg/dL glucose level. A water tank simulates the blood-glucose with 5 mm in depth, 5 mm in width and the dielectric constant $\varepsilon_{r_{w}}$ = 78.2.

Research by A. Kumar et al. [99]:

A. Kumar et al. proposed an LC-resonator-based biosensor to achieve a low resonating frequency system so that the penetration depth and the interaction areas are enhanced. Additionally, influencial factors like temperature were considered.

- *Methods*: The biosensor utilizes an LC-resonator. The applied inductor is chosen to be an intertwined air-bridge-type asymmetrical differential spiral inductor to improve the whole device inductance. The capacitor is a circular finger-type inter-digital capacitor with max. five circular fingers and a varying resonating frequency depending on the number of fingers. The system is proposed to have a resonating frequency of 1.5 GHz to accomplish a deep penetration according to Equation (4). Hereby, the value of penetration depth is calculated according to the deionized (DI) water. In consequence, a penetration depth of 34.46 mm is achieved.
  The detected S-parameters serve as the estimation factor to evaluate the accuracy of the fabricated sensor through comparison among the simulated, fabricated and DI water droplet sensors. Then, through the S-parameter and various glucose samples (30–500 mg/dL), the relation between resonating frequency and glucose concentration is found. After a linearization for calibration reasons, temperature effects are determined. According to the resonating shifts and the Q-factor shifts, a mathematical modeling of the sensor is developed, leading to the relation between the effective permittivity and glucose concentration.
  In addition, the glucose sample permittivity and relaxation time both affect the variation in complex permittivity, which can be observed by the Debye equations [98]
  (8) $$\begin{array}{cc} \underline{\varepsilon_{r}}\left(\omega \right) & =\varepsilon^{{}^{\prime }}\left(\omega \right)-j\varepsilon^{{}^{\prime \prime }}\left(\omega \right) \end{array}$$
  (9) $$\;\;\;\;\;\;\;\;\;\;\;\;\;\;\;\;\;\;\;\;\;\;\;\;\;\;\;\underline{\varepsilon_{r}}\left(\omega \right)=\varepsilon_{\inf}+\left(\varepsilon_{r,\mathrm{stat}}-\varepsilon_{\inf}\right)/\left(1+j\omega \tau_{\mathrm{relax}}\right),$$
  where $\varepsilon^{{}^{\prime }}\left(\omega \right)$ and $\varepsilon^{{}^{\prime \prime }}\left(\omega \right)$ are the real and imaginary part of the relative permittivity, $\varepsilon_{\inf}$ is the the permittivity at very high frequencies, $\varepsilon_{r,\mathrm{stat}}$ is the relative permittivity in the steady state, and $\tau_{\mathrm{relax}}$ is the relaxation time.
- *Results*: The proposed biosensor is able to detect glucose concentration in the range of 30–500 mg/dL in less than 5 s with an amplitude deviation of 0.004 dB/mgdL${}^{-1}$. In addition, the measurements were conducted at temperatures of 10–50 °C. The deviation of the tested and simulated accuracy-estimation is because of fabrication errors and impedance mismatching. Compared to the previously published glucose sensors, this sensor has a smaller size and low limitation of detection. Table 5 shows a summary of the results compared to the previous published studies, for example, utilizing Complementary Split Ring Resonators (CSRRs).
- *Further Improvements*: In the next step, the measurement should be performed under more realistic conditions, taking for example the human serum albumin with different age, gender and diabetes problems into account.

**Table 5:** Performance comparison of different resonator approaches.

Reference	Biosensor Structure	Concentration (mg/dL)	Size ($\lambda_{0}\times \lambda_{0}$)	Sample Amount (µL)	Sensitivity (dB/mg/dL)	Limit of Detection (mg/dL)
[99]	LC-Resonator	30–500	0.006 × 0.005	0.1	0.0049	35
[100]	LC-Resonator	25–500	0.026 × 0.060	1	NA	80
[101]	CSRR Resonator	30–400	0.251 × 0.386	NA	0.0003	NA
[102]	CSRR Resonator	0–500	NA	70	0.005	NA
[103]	Hilbert-shaped Resonator	50–250	0.408 × 0.808	500	0.000156	19.2

Research by A. Gorst et al. [104]:

- *Components:* The designed sensor is a coin-shaped near-field sensor, 25 mm in diameter and 0.76 mm in thickness. In addition, there is a VNA to measure the BGL and a hand model, which simulates biological tissue.
- *Methods:* The research is managed in three parts: model calculation, numerical simulation, and practical experiment. The blood considered here is venous blood. Therefore, instead of the various solutions in a container such as in the aforementioned studies, the solution is given through a silicon tube with a 5 mm inner diameter. In the hand model, the stratum corneum of the epidermis, the dermis, the subcutaneous adipose tissue, the hand vein and its wall and the fat are considered as having a thickness of 0.22, 0.04, 1.83, 1, 4, 0.5 and 6 mm, respectively. The system, including the hand model and the sensor, is operated in the frequency range of 0.5–5 GHz. Different saline solutions: 0, 18, 54, 72, 90, 126, 162 and 180 mg/dL (0, 1, 3, 4, 5, 7, 9 and 10 mmol/L) are simulated to get the relation between the real part of the dielectric permittivity and the frequency. In the practical part, the simulations are then validated in practice.
- *Results:* After applying the subtraction of the zero concentration to various concentrations, the maximum difference can be detected at around 1 GHz and in the range of 1.5–1.8 GHz. At the frequency of 1.07 GHz, the accuracy can be achieved with 0.1 dB in amplitude value of reflection coefficient $S_{11}$ to the glucose change of 18 mg/dL (1 mmol/L). However, in the practical experiment, the maximum difference is achieved at frequencies from 1.45 GHz to 1.55 GHz. The bias between the simulation (1.07 GHz) and the experiment is mainly caused by sensor manufacturing. In addition, the difference between the solution with 126 and 162 mg/dL (7 and 9 mmol/L) is hard to distinguish. In this range, at a frequency of 1.53 GHz, the sensor showed an optimal solution, which means the average variation in amplitude value of $S_{11}$ is 0.15–0.4 dB. However, the average value thereof is over the simulation result.
- *Further improvements:* In further research, the flexibility and mobility of the whole setup need improvements such as replacing the bulky VNA. Moreover, the time needed to perform the data collection and data processing should be reduced significantly.

Research by A.S. Zapasnoy et al. [105]:

- *Methods:* The proposed setup operates in near-field in the frequency range of 0.1 GHz to 10 GHz at 1 mm distance between the sensor and the tissue through the reflection coefficient $S_{11}$. There are two processing stages, namely, numerical simulation and experimental testing. The glucose level is monitored via the real part of dielectric permittivity. The sensor is conical, horn-shaped so that a wider passband can be achieved than in those with an open-end narrow-band probe. In the numerical simulation, conducted in the frequency range from 10 MHz to 10 GHz, biological tissues such as skin, blood, fat, muscles, and bones are considered. In the experimental testing, a sodium chloride solution with various contents of dextrose is used as a sample. The spectrum is analyzed with an N5230C power network analyzer (PNA-L) at its working frequency from 10 MHz to 40 GHz.
- *Results:* The simulation results illustrate the attenuation characteristics and penetration depth of different biological tissues. The penetration depths of blood, muscles and skin, of bone and of fat are 30, 60 and over 100 mm, respectively. In the experimental setup, when the frequency is lower than 1.5 GHz or over 3.5 GHz, the spectral behavior for different sample solutions cannot be distinguished. Therefore, the frequency range of 1.4–1.7 GHz is chosen. In this frequency range, the glucose concentration can be monitored from 0 to 450 mg/dL with a resolution of 1.8 mg/dL.
- *Further improvements:* The signal distinction at a frequency over 3.5 GHz can be improved, being not limited between 1.4 and 1.7 GHz.

## 3. Post-Processing

There are several methods to perform the data processing of glucose data, such as temporal abstractions, time series analysis and a combination of symbolic and numerical methods [106]. As the loss of data occurs quite often, interpolation and extrapolation are applied for more reliability. The processed data are then fitted into the regression equations. Such problems will be amplified, especially for individual glucose management.

The problem, however, can be dealt with by applying machine learning, which has drawn a great deal of attention for its advantageous data processing performance in optimization [107]. With CGM, the data recording is nowadays straightforward, and on the other hand, a huge amount of data are available. This large dataset is particularly advantageous for the machine learning method to improve the data analysis for better accuracy [32,35,108]. Notably, for the machine learning method, a large training set is needed, which means large biomedical data have to be available. However, the biomedical data are usually complex and disordered [109]. Thus, the pre-processing of the data is mandatory. The performance of machine learning depends not only on the algorithm itself but also on the similarity of the training set and the test set, which means the right choice of the dataset and the test set is essential.

Moreover, machine learning can even enhance the diagnosis as well as the therapy of diabetes by improving the prediction of BGL trends, thus reducing hyperglycemia and hypoglycemia [36,37,41,42]. Furthermore, the risk of getting secondary diseases can be estimated [39,40]. All in all, as machine learning has such a high potential for the post-processing of BG monitoring, in this paper, the chosen processing systems using machine learning methods for improving accuracy as well as predicting BGL trends are introduced.

### 3.1. Improving Accuracy via Post-Processing

Research by X. Xiao et al. [32]: 

The underlying sensing principle has already been introduced in Section 2.2. The samples under test are between 0 and 500 mg/dL. In one series, twenty-five data samples in the range of 20–500 mg/dL are collected with 20 mg/dL steps. These are later used as the training set. Additionally, five samples in the range of 50–450 mg/dL are taken with an interval of 50 mg/dL between each other, which serve as the test set. For each concentration, 255 different sets of data were investigated. All data from the training sets are used for *k*-fold cross-validation. The INNHO system applies two approaches, namely, the BPNN and LS-RANSAC. BPNN is used to find out the relation between the glucose concentration and S-parameters. After the training, LS-RANSAC is conducted on the estimated results to optimize estimation accuracy.

Before training, the data are pre-processed as follows: first, the difference between the measured S-parameter and that of water, the so-called relative S-parameter, is computed to minimize the system error; then, normalization is applied to have the frequency and the relative *S*-parameter in the same range [0,1].

BPNN is utilized for presenting the non-linear correlation among the frequency, the relative S-parameters and the glucose concentration, benefiting from a short processing time. The Broyden–Fletcher–Goldfarb–Shanno (BFGS) method is used in the training phase due to its 25% quicker calculation than gradient descent. The training phase will stop when the iteration reaches 400,000 times or the mean square error reaches 0.001. That means that the loss function is defined regarding the square error.

LS-RANSAC is used as a further accuracy improvement of the estimation. LS is widely used for fitting; however, it is sensitive to noise. That means, it has poor performance eliminating outliers. Therefore, RANSAC is conducted as a next processing step to decrease the influence of the outliers on the estimation accuracy of concentration.

**Figure 12:**
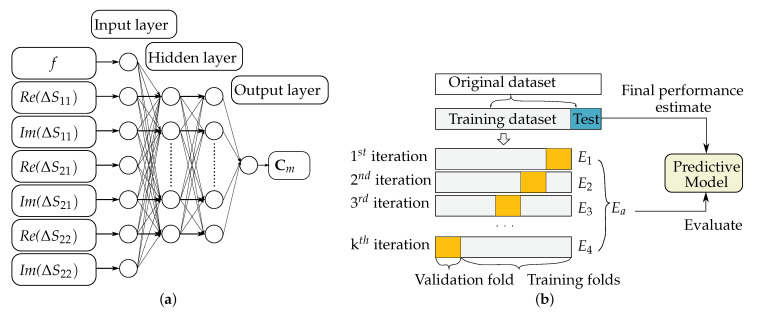
Overview of the signal post-processing redrawn according to [32]. (**a**) Structure of the applied neural network. (**b**) Structure of the applied cross validation.

In the network (Figure 12a), there are 7 input parameters, namely, the frequency *f*, the amplitude of the relative S-parameter (Re(ΔS11),Re(ΔS21),Re(ΔS22)) and the phase of the relative S-parameters (Im(ΔS11),Im(ΔS21),Im(ΔS22)). The hidden layer consists of 2 layers and 18 and 7 neurons, respectively. The training data set is built with 255×25×7 data. The output layer of the neural network outputs the blood glucose levels for every iteration. By executing the LS-RANSAC, the final concentration value is figured out. Finally, the *k*-fold cross-validation (Figure 12b) was applied, and the root mean square (RMSE) and the median absolute error (MAE) were calculated. Both the estimation accuracy with RMSE and MAE for 10 and 25 iteration times show a low evaluation metric being less than the ISO 15197 and the Food & Drug Administration (FDA) standard of 15 mg/dL and 12 mg/dL, respectively. The performance results are summarized and compared to the previously published studies [110,111,112,113] in Table 6. It is clear that the research by X. Xiao et al. is promising in terms of obtaining a lower RMSE of 5.53 mg/dL and a sensitivity of 0.0045 dB/(mg/dL) (equal to 0.07 dB/(15.48 mg/dL)).

### 3.2. Prediction of BGL Trends

Research by E.A. Pustozerov et al. [114]:

E.A. Pustozerov et al. elaborate on the so-called gestation diabetes mellitus (TGD), a particular type of diabetes [114]. The BG control effectiveness is evaluated through the postprandial glycemic response (PPGR), the prediction subject here. The decision tree gradient boosting algorithm is used for a prediction model, the xgboost model. This algorithm comes with a highly accurate prediction, even though some information about the BGL is missing, which means it still works promisingly even if some training data are lost. The loss function is the mean square error. The used data are meal-related, such as the amount of fat in the meal, the food context, such as the amount of carbohydrates consumed 3 h before the meal, and some patients’ personal characteristics, such as lifestyle, age, weight and height. In addition, an official mobile software is designed for the users.

PPGR includes the following characteristics:

- BGmax in mmol/L: The peak BG level after the meal started;
- iAUC120 in mmol/L·h: The incremental area under the glycemic curve 120 min after the meal start, which is the main factor in PPGR research;
- BGRise in mmol/L: The rise of BG from the meal start to the peak;
- BG60 in mmol/L: The BG value 60 min after the meal start.

Three ‘data’ scenarios are analyzed: without glucose measurement data before the start of the meal, a single data point measured with a glucometer or flash CGM, and with full CGM measurement data. That means, there are 12 characteristics. Further, there are some detection rules for eliminating incorrect or invalid information. For instance: More than half of the meals are recorded with the same dish.

The Pearson’s coefficient of correlation *R* and the MAE are utilized. In addition, the research team looked through various influence factors and chose the top three factors according to the Shapley value, namely, the meal’s glycemic load, the amount of carbohydrates, and the type of consumed food, especially at breakfast. The dataset with 3240 records of meals and corresponding PPGRs is separated into two parts: 75% thereof belong to the training set and the rest to the test set.

**Figure 13:**
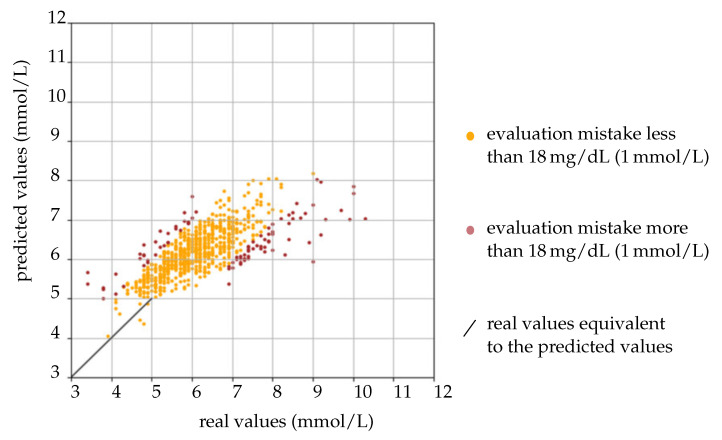
Correctness of real and predicted values of BGmax (Adapted from Ref. [114]).

The results of the BGmax model are illustrated in Figure 13: The yellow points represent a discrepancy between the predicted BGmax values and the real BGmax values of less than 18 mg/dL (1 mmol/L). The red points correspond to a variance larger than 18 mg/dL (1 mmol/L). It is quite clear that the number of the yellow points dominates the red ones, showing the reliability of the proposed method. In addition, compared to the previously published models, the work of E.A. Pustozerov et al. has a similar performance, which is summarized in Table 7.

D. Zeevi et al. [115] also aim to see the variability of food PPGRs but by applying gradient boosting regression, which predicts PPGRs with thousands of different decision trees. Their experiment was trained with 800 people and had two validation methods: leave-one-person-out cross-validation and independent 100-person validation. Additionally, H. Mendes-Soares et al. [116] aim to find the PPGR to food. The predictive model is based on D. Zeevi et al. [115] and uses the gradient boosting regression as well. However, E. A. Pustozerov et al. outperform both approaches, achieving R = 0.644 compared to 0.7 and 0.62 of the Zeevi et al. and H. Nedes-Soares et al.

In sum, the gradient boosting model offers promising prediction effectiveness and accuracy. However, since the patients’ meal data are mandatory, the reliability of this information has to be guaranteed, which needs further improvements, e.g., eliminating the incorrect information in the context of the documentation. Furthermore, the microbiome and metabolomics data should be taken into account.

Research by J. Martinsson et al. [117]:

Another approach was proposed by J. Martinsson et al. [117], which is based on recurrent neural networks (RNN) and achieves the BG level prediction up to one hour later. In the RNN, there are two hidden dense layers, called the dropout layer and the output layer. The dropout layer eliminates the overfitting data, whereas the output layer has two neurons, namely, the linear activation and the exponential activation. This characterizes a univariate Gaussian distribution.

The training set is based on the Ohio T1DM dataset [118], which contains an observation time of 8 weeks with a CGM record every 5 min, considering two male and four female patients aged 40 to 60. In addition, the dataset provides some additional self-reported information about the amount of consumed carbohydrates, exercise, sleep, and work. However, only the glucose records were evaluated because the authors want to prove that it is still feasible to predict the glucose level based only on them. Moreover, the dataset is divided into three parts, 60% of which serves as the training set, 20% for validation, and the rest for testing purposes.

Two loss functions are used, a negative log-likelihood loss function and a physiological loss function. However, the physiological loss function showed no improvement in the training phase. The applied optimizer, batch size, and the learning rate are Adam, 1024, and 0.001, respectively. For the model estimation, two criteria are used, namely, SEG and the RMSE. The final model is trained on the BG level with a duration of 60 min and predicts the BG level in 30 or 60 min. The results show an improved baseline.

### 3.3. Summary

As can be seen in the previous sections, post-processing using AI offers great opportunities in both, increasing the accuracy of measurement data and predicting the trends of blood glucose levels. The latter supports diabetic persons significantly, since it helps to avoid hyperglycemia and hypoglycemia. A comparison of different approaches with a prediction horizon of 30 min is given in the discussion summarized in Table 11. A more detailed table can be found in [37,42]. Further discussion regarding potential, challenges and concerns (used dataset, external validation) of AI in signal post-processing can be found in Section 5.2.

## 4. Commercial Devices and Systems

### 4.1. Commercial Devices

A commercial sensor must satisfy several criteria to get CE and Food and Drug Administration (FDA) approval, which implies satisfying the reliability, consistency, and safety criterion [77]. The corresponding accuracy for the EU, defined by the European Medicine Agency (EMA), and for the USA, defined by the FDA, are listed in Table 8. The FDA requires that 95% of all measurement values should be for BGL ≥75 mg/dL within the range of ±12% compared to the reference values and, additionally, for BGL <75 mg/dL within ±12 mg/dL. Furthermore, 98% of the measurement results should not exceed ±15% for BGL ≥75 mg/dL, and ±15 mg/dL for BGL <75 mg/dL, respectively [119].

For determining the accuracy, there are several methods such as the mean absolute relative difference (MARD), root mean square error (RMSE), correlation coefficient, systematic measurement difference (bias) and error grids (namely, Clarke-, Consensus- and Surveillance error grid) [37,121]. In the proposed paper, the accuracy of state-of-the-art non-invasive glucose sensors and commercially available sensor systems will be further discussed (next to the already introduced metrics) using the Clarke error grid.

**Figure 14:**
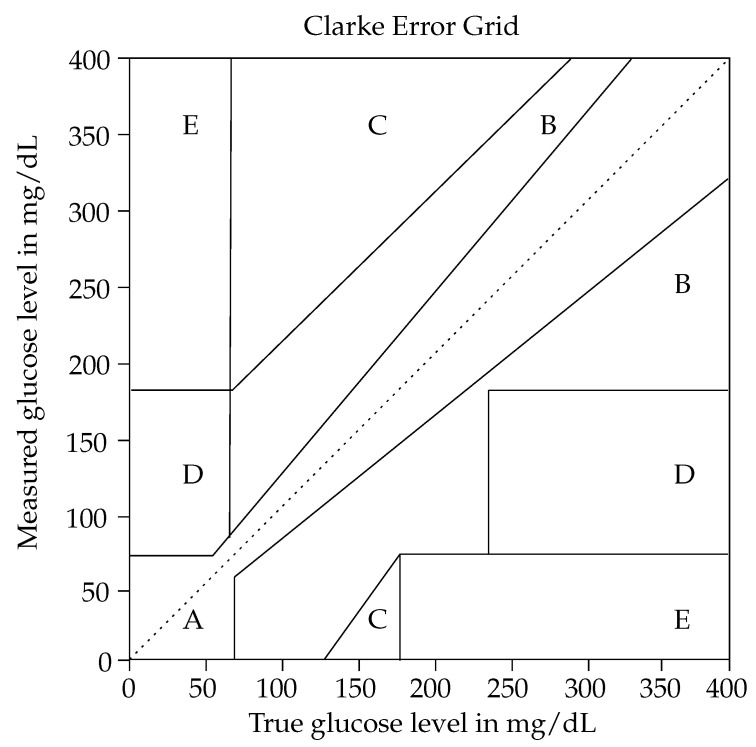
Clarke error grid model. The region A shows the desired accuracy of a glucose sensing system to fulfill clinical accuracy requirements.

According to this model, the accuracy of a glucose monitoring system has to meet strict requirements [122]. The Clarke error grid compares the true BGL with the measured BGL and is illustrated in Figure 14. If the measured BGL perfectly fits the reference (ideally true) BGL, it is located on the bisector of the grid. The more the true and the measured values differ, the more dangerous it can be for the patient. This is represented by the different zones A-E in the grid. A glucose sensor is classified as a clinically valid treatment when the tolerance of the glucose level is below 20% (see region A in Figure 14), or both the true and the measured BGL are below 70 mg/dL, since the latter corresponds to hypoglycemia. The other zones, B, C, D and E, correspond to clinically uncritical treatment, unnecessarily treatment, dangerous fails to diagnose and treat, and extremely dangerous leading to wrong treatment, respectively [121].

In addition, the success of a commercial device is not only about the technology but also about the cost. In consequence, various approaches using different sensor principles are proposed by different companies, e.g., the TensorTip Combo Glucometer by Cnoga Medical Ltd (NI-optical, CE Mark, not cleared by FDA), the sugarBEAT by Nemaura Medical (NI-fluid-based, CE Mark, not cleared by FDA), and Eversense by Senseonics (minimal invasive, CE Mark and FDA cleared) [123]. A detailed review was published by Shang et al. [123] (2021). They investigated in total 65 different blood glucose monitoring products with different statuses of development regarding their advantages and disadvantages. The products include 28 non-invasive optical products, 6 non-invasive fluid sampling products, and 31 minimally invasive products. Few of the sensor systems have received the CE Mark and/or have been cleared by the FDA yet. Some of them were discontinued, such as the GlucoWatch Biographer from Cygnus Inc. and the Pendra Device from Pendragon Medical because of issues about burning sensation and inaccuracy, respectively, or did not enter the market, such as the NBM-200G from OrSense [26,77,108,123]. Although there are many failed commercial devices, others are successful, such as the two popular commercial systems, FreeStyle Libre and Dexcom. Table 9 gives a brief comparison of both. The price for both the FreeStyle Libre and the Dexcom, approx. 60€ [124,125] each plus additional costs for a reusable transmitter/reader.

### 4.2. Commercial System

Both Freestyle Libre and Dexcom have released several generations. The well-known versions are the second Freestyle Libre generation and the sixth generation of Dexcom. In the following, a more detailed overview of different generations of Freestyle Libre and Dexcom is given.

FreeStyle Libre has already released three versions. Those three versions are all CGM devices. For the first FreeStyle Libre generation, there is no acetaminophen interference and no calibration, and it is inaccurate in indicating hypoglycemia. In detail, 40% of the time a BGL under 60 mg/dL is reported, whereas the actual BG value is in the range of 81–160 mg/dL [127,128,129,130]. Moreover, the inaccuracies occur on the first and last days of the 14 days working time with a MARD of 11.2% [131].

**Figure 15:**
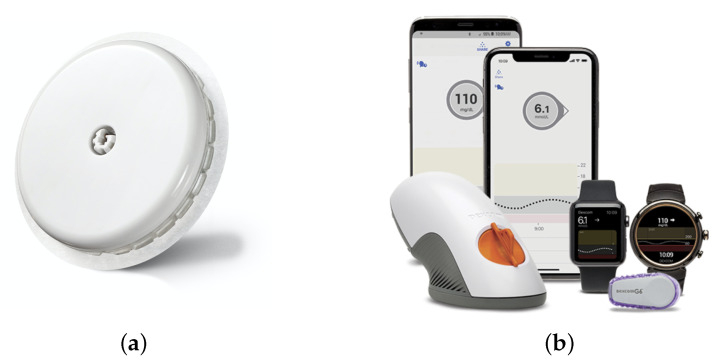
Commercial systems of FreeStyle Libre 2 and Dexcom G6. (**a**) FreeStyle Libre 2 sensor (Reprinted with permission from Ref. [135] *©* Abbott GmbH). (**b**) Dexcom G6 sensor (Reprinted with permission from Ref. [136] © Dexcom, Inc).

Meanwhile, the second version was released in 2020, the so-called FreeStyle Libre 2 [126]. The sensor of FreeStyle Libre 2 (Figure 15a) provides information about the continuous measured blood glucose level, optional results for finger pricking, and a prediction of a rising or falling trend. Especially, the trend helps not only the patients but also the medical specialists to manage the blood glucose level. In addition, there exists a specific FreeStyle LibreLink APP to assist the user. Like the first generation, the sensor works for 14 days in the range of 40–500 mg/dL, and it is small in size and comfortable to wear, being 5 mm in height, 35 mm in diameter (see Figure 15a), 5 g in weight and worn at the backside of the upper arm [126]. The cost of FreeStyle Libre 2 was analyzed by I. Oyagüez et al., who showed that about 43.1% is saved compared to self-monitoring of blood glucose (SMBG) [132].

FreeStyle Libre 3 was released in 2021 [23]. It is designed for children from the age of 4 onwards, works for 14 days, and is still worn on the backside of the upper arm [23]. For the data reading, the personal mobile phone via Bluetooth is used instead of a separate reading device. The BG monitoring range is 40–500 mg/dL, and the size is 2.9 mm in height and 21 mm in diameter, while the weight is stated to be 1 g.

A competitor of FreeStyle Libre is Dexcom with its BG sensor technologies. Dexcom has several versions, like G5 and G6. Dexcom G7 is in development [133] and the first study results were already published [134]. Dexcom G6 consists of three parts, namely, an auto-applicator, a sensor and transmitter, and a display device [22]. All components with different display devices are depicted in Figure 15b. It works for 10 days. Compared to FreeStyle Libre 2 and 3, Dexcom G6 has three possible sensor positions: belly, the back of the upper arm and upper buttocks. It is also suitable for children from the age of 2 onwards [22]. As can be seen in Table 9, the G6 is significantly heavier than the FreeStyle Libre sensors; however, the G7 is expected to be reduced in size by about 60% [133].

## 5. Discussion

Diabetes is a chronic disease. More precisely speaking, in case of T1D it is until now an incurable disease. The glucose level must be within a specific range to prevent further damage to a patient by avoiding hyperglycemia and hypoglycemia. Therefore, a glucose monitoring system should continuously track the glucose level with high accuracy (<20%). Thus, research in glucose monitoring has attracted attention for years, from the early conventional diagnosis to intensified diagnosis, which nowadays, it is desired that it be non-invasive. Meanwhile, many commercial devices were in the market to provide a reliable glucose measurement. However, most are unsuccessful in receiving the FDA or CE approval or are discontinued afterwards. At present, two commercial systems are dominant in such a field, namely, FreeStyle Libre and Dexcom. In recent years several generations have been released. The latest versions are FreeStlye Libre 3 and Dexcom G6. The advantages of these systems are that both provide continuous glucose measurement and work for at least ten days, which eases the burden of glucose management on both patients and medical specialists. Nevertheless, both commercial systems belong to minimal-invasive technology. Therefore, the risk of infection, pain for the patient and contact allergy and the cost of sensor replacement are still existing disadvantages.

To satisfy the demand for less costly, more convenient, and more accurate glucose measurement and monitoring devices, much research is conducted on non-invasive monitoring technology. In the proposed review, recent electrochemical-based and electromagnetic-based measurement systems are introduced and compared. An overview of exemplary state-of-the-art approaches is listed in Table 10. Furthermore, the opportunities and challenges of advanced post-processing are discussed, and finally, all proposed approaches are characterized and compared using the Clarke error grid.

### 5.1. Non-Invasive Sensor Principles

For electrochemical-based measuring of the glucose level, different biological medium such as saliva, tears, exhaled breath and blood are regarded as the MUT. However, many distortion factors (e.g., foot or beverage intake) exist in saliva, tear and exhaled breath, as the molecules inside are various and complex. Therefore, an additional layer was proposed as a standard solution to such a problem, although the function is different. The authors of [29], added a membrane to filter the molecules inside the saliva to increase the glucose measurement accuracy, based on the design in [51]. Similarly, an additional layer with different supporting polymers was proposed in [58] or for condensation of VOCs in [73]. The difference is that such a layer is not for filtering but for sensing. Especially, sensing using exhaled breath is regarded as controversial in the discussion regarding the correlation between BGL and measured acetone because of the significant influence of various factors. In consequence, exhaled breath is currently commercially investigated for diet management or diabetes diagnosis and not as a CGM sensing system [73,74,75]. However, for example GLUCAIR™ is currently working on a commercial solution for non-invasive blood glucose monitoring using exhaled breath [43]. In general, secondary liquids, such as saliva, urine, teardrops or interstitial fluid, face the problem of latency compared to glucose variations in blood. For example, the duration of the glucose observation in [29,58] is about 20 min. The requirement for CGM is, however, a short latency between the measured and real glucose value. Additionally, the biosensor material must be waterproof and manufactured to be biocompatible, which is crucial for avoiding skin irritation or rejections but increases the costs of a biosensor.

Blood is more commonly used than saliva and tears as MUT for electromagnetic-based glucose sensors. In [83,84,137], Raman Spectroscopy was applied. A linear relation was observed between the signal intensities and the glucose concentration difference in [83]. The minimal detectable change was reported to be 29–78 mg/dL, which is not suitable for real application. In [141], a non-invasive Raman spectroscopy sensor was proposed. The sensor was placed on the microvessels of the nail fold. A mean accuracy of approx. 8.1 mg/dL was reported on 12 subjects. Although the results for optical-based sensing systems of [141] and of [142], where 35 patients were evaluated, were located mainly in Zone A and B of the Clarke error grid, the strong dependency on the temperature and environmental aspects, such as scattering light, has to be mentioned. Moreover, their relative bulky structure makes it difficult to integrate into a wearable glucose sensing system.

The impedance spectroscopy-based glucose sensors with operating frequencies in the kilohertz to megahertz range, have been extensively explored by researchers over the past years [85,87,88,89]. This effort resulted in a shortly commercially available impedance spectroscopy-based glucose monitoring system called Pendra. However, post-market studies revealed that the accuracy partially fell in the dangerous Zone C of the Clarke error grid, and thus, it was removed from the market. The impedance of human tissue is affected by several factors such as temperature, sweat, skin thickness and moisture, which vary over the day and also from patient to patient. Moreover, since electrodes are placed on the skin, relative movement between the skin and the electrodes may change the measured impedance as well as have the potential to lead to allergic reactions. To increase the overall accuracy and stability of impedance spectroscopy-based glucose sensing, recent studies combined those sensors with multiple sensors, such as temperature, humidity and optical ones [87,89] and also with accelerometers [89]. Thereby, promising results were achieved in in vivo experiments. Moreover, the time lag between the physiological parameters and the estimated glucose values was reduced by using time series analysis and sensor fusion [87]. However, the study population of 9 [87] and 20 [89] was still relatively small and in [89] 12.1% sensor readings fell in the potential dangerous Zone D of the Clarke error grid. Therefore, impedance spectroscopy-based sensors are promising and yield the potential to be integrated into a wearable sensors system to enhance the overall performance and stability of non-invasive glucose sensing in the daily life situations of a patient.

The microwave-based approaches to miniaturization show a quite promising performance with low fabrication costs. The sizes of the sensors are in the range of centimeters to millimeters and even go down to the micrometer range, such as in the case of A. Kumar et al. [99], with a sensor of 0.3 mm × 0.25 mm. Consequently, these sensors are highly applicable to wearable systems for a patient’s daily life. However, one of the most crucial design criteria is getting the measurement signal to the area of interest (blood vessels) through the different tissues (e.g., skin, fat) and back to the sensor, respectively. If the signal levels are too low, changes in BGL will not be detectable. This significantly depends on the chosen operating frequency, which defines the penetration depth. The working frequencies are mostly in the range of 0–6 GHz [32,92,95,96,97,99,104,105,138,139,140], and the others are in the range of 10–20 GHz [93,98]. In general, the higher the frequency, the lower the penetration depth. Considering their similar working frequencies and working principles, the performances of [32,99] and [104] are similar, with accuracies of 0.0045 dB/(mg/dL), 0.0049 dB/(mg/dL) and 0.003 dB/(mg/dL), respectively. For the other working frequency range, [98] works better than [93], as the phase change to the glucose change is larger to 2° per 10 mg/dL. In addition, different from other approaches, [97] introduced sub-band processing. V.V. Deshmukh et al. proposed different frequency bands for diabetes situations (with diabetes, without diabetes and pre-diabetes) to increase the accuracy.

In addition, the choice of the antenna and its corresponding tapering are essential as this will also define the available signal strength. X. Xiao et al. [32] utilized an UWB-antenna, whereas V.V. Deshmukh et al. [96,97] recommend narrow-band antennas since they provide a more accurate linear response between frequency and BGL.

In general, the proposed scientific microwave approaches are in different development stages: conducting only simulations or developing a corresponding (mathematical) simulation-based model and validating this with experiments under ideal and under realistic conditions. Besides this classification, the scientific approaches can also be divided into those that only investigate the sensors system and those that utilize advanced signal processing for improving sensor accuracy. Depending on the desired mounting position, several tissue models were analyzed. X. Xiao et al. [32] applied an earlobe model, whereas A. Gorst et al. [104] designed a detailed hand model considering the complex structure of the different tissue layers. Other researchers prefer to use the fingertip for measuring the BGL, since a low penetration depth is needed to reach the intra vascular blood in the micro vessels of this region [35,140]. Fingertip approaches provide great prospects of success, since they outperform systems using other detection areas. These are mostly resonator-based such as in the cases of Omer et al. [35] or Kiani et al. [140]. However, they are based on placing the fingertip on the sensor area, and therefore, they can be a good alternative to finger pricking but are not suitable for a CGM system.

Furthermore, the proposed methods differ in the evaluated liquid. Most approaches use water with glucose or dextrose solutions [98,99,104], whereas a minority conduct measurements with real blood in the lab or even with humans. M.Hofmann et al. [93] mixed real blood with NaCl and water, whereas V.V.Deshmukh et al. [96,97] conducted measurements with a study population consisting of non-diabetic, pre-diabetic and diabetic persons.

In the literature, there are three methods discussed for detecting the change of the permittivity and the corresponding BGL: shift of resonance frequency [99,100,101,102,103], reflection ($S_{11}$) [32,96,98,104,105,111] or transmission ($S_{21}$) [32,110,112] of the amplitude or phase of the *S*-Parameters. S. Zeising et al. [98] stated that the phase variation of $S_{11}$ is more sensitive than of $S_{21}$. X. Xiao et al. [32] utilized both, $S_{11}$ and $S_{21}$, plus advanced signal processing improving the performance of RSME to 5.53 mg/dL. Modern signal processing techniques like machine learning can improve the performance of the BGL-detection significantly.

### 5.2. Post-Processing

Overall, post-processing using AI offers excellent opportunities to increase the accuracy of measurement data and predict the trends of blood glucose levels. The latter supports diabetic persons significantly since it helps to avoid hyperglycemia and hypoglycemia. However, in the prediction, there is a trade-off between accuracy and the prediction horizon [37]. This gets particularly interesting for predicting, warning and thus avoiding hypoglycemia during sleep, which can be highly dangerous for the patients [143,144]. A comparison of different approaches with a prediction horizon of 30 min is given in Table 11.

For the validation, a trustworthy reference BGL-value is indispensable, which is carried out e.g., via the invasive finger pricking [97] with a commercial system or with a bulky and expensive VNA [32,95,104]. Both options are not satisfactory for a future minimal-invasive system solution. J. Martinsson et al. [117] used the available Ohio T1DM dataset [118], which collects the CGM record of 6 persons, aged 40 to 60, every 5 min over 8 weeks to predict BGL trends. The approach of J. Martinsson et al. [117] is based on RNN. The final model is trained with a duration of 60 min and predicts the BGL in 30 or 60 min. This is applicable for commercial systems, such as for example, the FreeStyle Libre 3, which also has a calibration time of one hour. E.A. Pustozerov et al. [114] structured their dataset meal-related considering the food context and patients’ personal characteristics. For signal processing, they used an xgboost model (boosted decision trees) and were able to achieve R=0.644. However, for a more realistic, daily-life study, the microbiome and metabolomics data should also be considered. D. Zeevi et al. [115] trained their model with 800 persons and considered microbiomic features. However, their performance was worse compared to [114] with R=0.70.

A potential way to increase the performance of prediction is by taking additional influence factors such as temperature into account. This can be carried out by sensor fusion [52,87,157]. For example, Geng et al. [87] were able to improve their results significantly by combining the impedance spectroscopy-based glucose sensing system with temperature, humidity and optical sensors. However, it is worth mentioning that considering too many additional sensors can lead to misinterpretation and an increased noise level. Thus, only the essential features should be evaluated.

Moreover, the results of AI in general strongly depend on the dataset and the model design (training, validating, and testing). Next to unrecognized biases in the dataset, the accuracy of the results can be distorted by a wrong validation. Thus, appropriate testing combined with external validation is crucial. However, the available datasets are often limited by a small-sized samples or missing data regarding reference BGL or features such as eating, physical activities or insulin injection. In consequence, generalization is a problem. Additionally, reproducibility is a fundamental problem, as only few researchers share their codes and/or datasets [33].

Overall, AI-based post-processing offers opportunities such as being able to predict BGL trends, and furthermore, it outperforms traditional signal processing approaches by enhancing the sensitivity of sensor systems. For the approaches using AI to increase their sensor performance validation is critical. For the required reproducibility, the study design must be defined for all the influential factors such as conditions of the sensor itself (hardware, fabrication errors), measurement environment (temperature, humidity), generalizability of tested persons (gender, age, type of diabetes) and algorithm.

Indeed, X. Xiao et al. [32] fulfill the ISO 15197 and the FDA-standard by using BFGS for training; LS-RANSAC for optimization and BPNN for presenting the correlation between the frequency, the *S*-parameters and the glucose concentration. However, they do not use patient related data, which makes their work not comparable to others. Omer et al. [35] were able to further enhance their measuring results by applying the PCA feature extraction algorithm and a one-time personalized invasive calibration to identify the blood glucose level patterns from the fingertip. The in vivo measurements were conducted with one healthy 29-year male volunteer for about one minute. The measured BGL was validated with finger pricking. Here, the dataset with only one tested person was insufficient for external validation and could be seen more as a proof of concept. In sum, the reviewed literature has gaps related to many of the aforementioned points regarding reproducibility and generalizability since many of them are not mentioned/considered. Thus, it remains an open question whether these systems are externally validated in full.

### 5.3. Evaluation with Clarke Error Grid

Based on the different levels of development of the proposed sensor systems in the literature, the archived accuracy varies significantly. Several researchers demonstrated only a proof of concept of their approach, by simulating [98] and measuring [92,93,95,104,105] physical parameters such as $S_{11}$ or $S_{21}$. All of them showed that the amplitude and phase as well as the resonance frequency depend on the glucose concentration; however, a specific determination on their measured BGL was missing. In consequence, their methods cannot be evaluated by means of the Clarke error grid.

An overview of the other approaches regarding the Clarke error grid is listed in Table 12: The sensor readings of commercial glucose monitoring systems fell, particularly in zones A and B. However, the results of [158,159,160] revealed that up to 2.1% of the sensor readings were in the dangerous zone D.

By applying Raman spectroscopy, 93% of the sensor readings fell in zones A and B [84]. However, the study was only simulation-based. Moreover, in [141], 100% of the sensor readings fell in zones A and B in measurements on twelve subjects. Compared with commercially available monitoring systems, this is a good performance. Raman spectroscopy-based sensors are relatively bulky and expensive. Therefore, they are not suitable for a wearable glucose monitoring device considering the state of the art. Moreover, in [142], photoplethysmography (PPG) was used for non-invasive glucose sensing. The reported accuracy was slightly worse than that of commercial sensors with 96.85% in zones A and B and with 3.15% in the dangerous zone D. The limitation of this study was that heterogeneous finger models were considered. Nevertheless, the thickness of the tissue layers can vary from person to person.

**Table 12:** Overview of the accuracy according to the Clarke error grid. Abbreviations: P = Persons, T1D = diabetes mellitus type 1, T2D = diabetes mellitus type 2, TGD = gestational diabetes mellitus, NA = Not a Number.

Reference	Measuring Method	Detection Range in mg/dL	Dataset	Clarke Error Grid:				
				A	B	C	D	E
**Commercial Sensor Systems:**								
[158]	Dexcom G6	40–400	25P T1D (resistance), 30 min each (aerobic), 30 min each	85.4%	12.5%	0%	2.1%	0%
				74.0%	26.0%	0%	0%	0%
[159]	FreeStyle Libre	30–400	24P T1D, 11P T2D, 39P TGD, all pregnant, 4207 data points	83.6%	15.5%	0%	0.8%	0%
[160]	FreeStyle Libre	40–500	30P T2D, 1353 data points	88.54%	11.01%	0%	0.45%	0%
**Optical Sensor Systems:**								
[84]	Raman Spectroscopy	50–400	10.000 synthetic generated spectra	93.0%		NA	NA	NA
[141]	Raman Spectroscopy	105–216	30 meas. × 12P	100%		0%	0%	0%
**Microwave-Based Sensor Systems:**								
[97]	Microwave	60–400	205P without categorization	80.91%	19.09%	0%	0%	0%
			205P with categorization	95.12%	4.88%	0%	0%	0%
[99]	Microwave	30–500	6×6 meas.	100%	0%	0%	0%	0%
[104]	Microwave	0–180	7×10 meas.	85.7%	14.3%	0%	0%	0%
[139]	Microwave	0–400	10 min in total, 2 min each, concentration level (CGM)	100%	0%	0%	0%	0%
			5×6 meas. for:					
[161]	Microwave	50–500	1. Silver-painted device	44.45%	40.74%	3.70%	11.11%	0%
			2. Adhesive copper tape device	68.97%	24.14%	0%	6.89%	0%
**Sensor Systems with Advanced Post-Processing:**								
[32]	Microwave Post Processing INNHO	20–500	255×5×7 data points	100%	0%	0%	0%	0%
[87]	Impedance Spectr./Sensor Fusion Post-Proc.: Time Series Analysis	0–200	3 T1D P and 6 healthy P	100%		0%	0%	0%
[114]	Flash CGM Post Processing xgboost model	60–180	198 TGD, 37 healthy P 3240 data points	100%		0%	0%	0%
[117]	Medtronic Enlite CGM sensors Post Processing RNN	30–400	Ohio T1DM dataset [118] 25142791 data points	patient dependent, >90% in A and B				
[142]	Photoplethysmography (PPG)	50–150	synthetic	80%	20%	0%	0%	0%
	Monte Carlo Simulation	80–200	real data (35P)	91.8%	5.05%	0%	3.15%	0%
[146]	RNN	30–400	Ohio T1DM dataset	90%	9%	0%	1%	0%
[148]	Grammatical Evolution (GE)	30–400	Ohio T1DM dataset	87.1%	11.5%	0%	1.4%	0%

Many scientists are working on the development of microwave-based non-invasive glucose sensors. Gorst et al. [104] proposed a parabolic regression between the glucose concentration and $S_{11}$, whereas Kumar et al. [99] obtained a linear relation. However, A. Gorst et al. [104] considered a larger glucose range from 18 to 180 mg/dL compared to Kumar et al. [99] with concentrations up to 90 mg/dL. Based on these regressions, an evaluation regarding the Clarke error grid can be carried out: Gorst et al. [104] were able to achieve all results in A apart from one in B; the results of Kumar et al. [99] are highly accurate (all in A) and reproducible. However, both of them evaluated only a few data points. Deshmukh et al. [97] conducted the measurements on 205 persons containing non-, pre-, and purely diabetic people. In the Clarke error grid, they predicted only BGL in A or B (A=80.01 and B=19.09%) without a categorization. By using different frequency bands according to the diabetic classification, their results improved to 4.88% in B, and the results in A were also closer to the real values. Moreover, X. Xiao et al. [32] were able to improve their results significantly by post-processing the data using the INNHO method. Without the INNHO method, the data points were distributed all over the Clarke error grid; even in the extreme risk zone E. By applying the INNHO method, all values were categorized in A apart from one for 50 mg/dL in D. Similar results proposed Pustozerov et al. [114], having also outliers in D for BGL <70 mg/dL. However, Martinsson et al. [117] stated that the performance of the BGL diagnosis also strongly depends on the individual person. For one person, nearly all values could be classified in A, whereas the results of another person with the same RNN had also values in the high risk zone D. Nevertheless, due to the small number of test persons (6), the reason for the deviation was not further investigated. In addition, resonator-based measurement systems were proposed by Jang et al. [139] and Juan et al. [161]. In [139], 100% of the sensor readings fell in zone A, whereas in [139] up to 93.11% were in zones A and B and 6.89% in zone D. Therefore, the results of [139] outperformed commercial sensors. However, the performance of the resonant-based sensor was evaluated with a DI water solution, which does not represent real conditions for non-invasive glucose sensing.

In addition, Table 12 shows the results of an impedance spectroscopy-based sensor combined with multiple different sensors [87]. Herein, 100% of the sensor readings fell in zones A and B, which is considered as clinical accuracy. The results were significantly improved by adding humidity, temperature and optical sensors to the wearable system and applying time series analysis as post-processing. However, the study population was relatively small, with nine subjects. Furthermore, the observed detection range was insufficiently limited to 0–200 mg/dL. According to the requirements of the FDA, an appropriate sensor system used outside the hospital must be able to detect the BGL in a range of 20–500 mg/dL [119]. Most of the systems proposed in Table 12 cover this required range approximately. However, approaches such as that of [104,114,141,142] are also limited in their analyzed detection range. The Clarke error grid results of those have to be interpreted with caution, since it is easier to design a precise sensor system for a narrow detection range than for a broad one.

In sum, the microwave approaches show the most promising results regarding miniaturization and low fabrication cost. Moreover, the accuracy of those approaches is comparable to that of commercial sensors. Kumar et al. [99] achieved a microwave-based sensor that is independent from temperatures between 10 and 50 ${}^{\circ }$C. However, they conducted their measurements with a DI water drop, and therefore, it is questionable if the temperature independence is also fulfilled for a blood drop. Furthermore, using a drop of blood is invasive and, thus, cannot be seen as an improvement compared to traditional finger pricking. In addition, in the microwave-based approaches, the sensor must have direct contact with the skin, with no air gap in between, since the impedances of the sensors are designed to match those of the skin. Impedance matching is the most critical point in measurement scenarios, especially in daily life, since it also depends on immutable factors such as the temperature and the humidity of the skin or the sensor mounting compression. If the sensor loses contact with the skin, there is a jump in the impedance, which leads to high losses, and thus, the change of the BGL is no longer detectable. The impedance spectroscopy-based sensors suffer from the same problem. Since electrodes are fixed on the skin, relative movement between the skin and the electrodes results in an impedance change. Moreover, the impedance of the tissue is affected by the temperature, sweat or moisture level of the tissue. Therefore, they are usually combined with other sensors. The optical-based sensors are in the early stage of development and are not suitable for a wearable system due to their bulky size. Moreover, optical-based sensors significantly depend on the temperature. Overall, the accuracy of scientific solutions for non-invasive glucose monitoring is comparable with commercial sensors. Nevertheless, it is worth mentioning that gold standard finger pricking—which is often used as a reference value for sensors characterization [87,97,140,162]—also does not have 100% of the measurement results in Zone A of the Clarke error grid as shown in [163].

In future scientific approaches to non-invasive glucose sensing systems, additional criteria (apart from the sensor sensitivity, safety and accuracy), such as sensor size, battery lifetime and convenience of the sensor should be considered. Since nowadays it is common to track the fitness level via a smart watch, a glucose sensor is proposed to be assembled in the electric watch instead of as a separate device, such as Apple is planning to do with the Apple Watch [164]. On the other hand, some new approaches are underway through cell therapy with β-cells [165]. Some companies are actively involved, e.g., Sernova, which is a regenerative medicine company, is developing new therapeutic technologies. Recently, they proposed Sernova’s Cell Pouch System™, which is an implantable and scalable medical device [166]. Additionally, a clinical trial, the so-called functional cure, is now in process, which captured a great deal of attention at the beginning of the year [167].

## 6. Conclusions

The lives of those who face diabetes differ significantly from those of non-diabetics. Patients must test their blood glucose levels at least several times a day. Although people nowadays can use insulin pumps with integrated blood glucose sensing systems as an automatic way to monitor and control their blood glucose, there is still an increased risk of infection. That is why non-invasive methods, such as those using saliva, tear, or electromagnetic-based sensors embedded in wearable devices, are attracting increased attention. Since electromagnetic sensors offer several advantages, such as low fabrication cost and independence in terms of temperature, they are the most promising approaches to non-invasive blood glucose monitoring. However, they are highly sensitive to penetration depth, operating frequency and tapering. On the other hand, artificial intelligence is gaining importance in signal processing to improve accuracy and predict the development of the blood glucose level precisely. Moreover, combining microwave-based sensors with multiple sensors such as temperature, humidity, or impedance spectroscopy-based sensors could improve the overall accuracy and stability of a non-invasive glucose sensing system. In this paper, the state of the art is reviewed, focusing on comparing scientific electrochemical and electromagnetic non-invasive approaches to already existing commercial solutions. A summarized overview of the various approaches is given in Table 10. Companies such as Sernova are introducing novel diabetes therapies, whereas sensors like the ones of FreeStyle Libre or Dexcom are already commercially available and are being steadily enhanced. However, considering the current situation, under the COVID-19 pandemic, a reliable, low-cost blood glucose monitoring sensor enabling tele-medical care is in high demand. Thus, there is still substantial room for improvements in terms of better accuracy, stability, safety, efficiency, simplicity, lab-on-chip compatibility, and miniaturization.

## Figures and Tables

**Figure 11:**
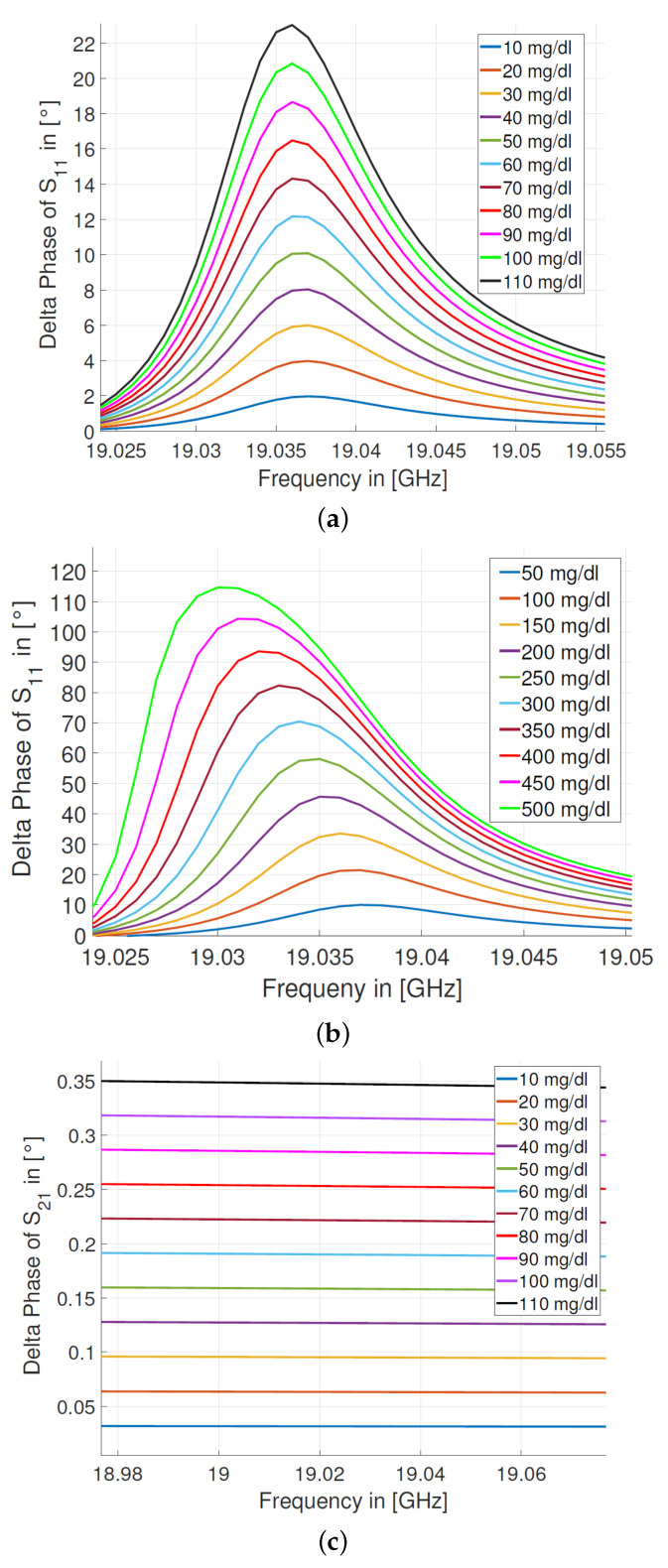
Phase variation of $S_{11}$ and $S_{21}$ due to the different glucose concentrations (Reprinted with permission from Ref. [98] © 2020 IEEE). (**a**) Phase variation of $S_{11}$ with a glucose concentration of 10–110 mg/dL. (**b**) Phase variation of $S_{11}$ with a glucose concentration of 50–500 mg/dL. (**c**) Phase variation of $S_{21}$ with a glucose concentration of 10–110 mg/dL.

**Table 1:** Response characteristics of each supporting material according to the results of [58].

Supporting Material	Limit of Detection (LOD)	Maximal Reaction Rate ($V_{\max}$)	Michaelis–Menten Constant ($K_{m}$)
EC	0.5 mg/dL (0.025 mM)	8568 mg/dL·min (476 mM/min)	5.1 mg/dL (0.286 mM)
D4	0.5 mg/dL (0.029 mM)	2534.4 mg/dL·min (140.8 mM/min)	6.6 mg/dL (0.366 mM)
GA	0.8 mg/dL (0.043 mM)	1314 mg/dL·min (73 mM/min)	6.6 mg/dL (0.364 mM)

**Table 2:** Reversibility performance and comparison of SI for standard and tear glucose with the three supporting materials EC, D4 and GA in glucose according to the results of [58].

Supporting Material	RSD at 0 mg/dL	RSD at 36 mg/dL	${\mathrm{SI}}_{\mathrm{standard}\;\mathrm{glucose}}$	${\mathrm{SI}}_{\mathrm{tear}\;\mathrm{glucose}}$
EC	0.32%	0.15%	0.0555	0.0503
D4	0.38%	0.79%	0.0821	0.0846
GA	23%	0.33%	0.0561	0.057

**Table 3:** Prediction evaluation results of the three criteria. R: regression analysis, MARD: mean absolute relative difference and CEG A + B: zones A and B in the Clarke error grid according to the results of [84].

Prediction Method	($\eta_{\mathrm{collect}}$ Five Times Halved from 3.2% to 0.2%)		
	R	MARD	CEG A + B
RF regression	0.91 → 0.35	20.3% → 54.6%	93.0% → 82.4%
PLSR	0.91 → 0.34	20.3% → 54.8%	93.0% → 82.4%

**Table 6:** Performance comparison of recently published earlobe models, concerning analytical as well as neural network approaches such as Complex-Valued Neural Networks (CVNN). Cal. corresponds to calibration.

Reference	Frequency (GHz)	Utilized Data, Calibration	Estimation Method	Dataset	Concentration (mg/dL)	Sensitivity (dB per mg/dL)	Performance
[110]	60	$S_{21}$ No Cal.	Analytical	10 healthy men in vivo	23.94–4788	$0.65\times 10^{-3}$	Glucose spike monitoring test
[111]	1.4–1.9	$S_{11},Z_{11}$ Cal. not mentioned	Data Fitting	12 meas. samples in vitro 5000 pseudo samp.	78–625 625–5000	$1.8\times 10^{-3}$ $6.6\times 10^{-3}$	Average error 20 mg/dL 50 mg/dL
[112]	60–80	$S_{21}$ Cal. not specified.	CVNN	7×30 meas. sampl. in vitro	50–300	—	Estimation for 100 mg/dL: 80–107 mg/dL internal validation
[113]	3–10	Absorption Spectrum Cal. not mentioned	Linear Fitting	meas. from 0 to 500 mg/dL in step of 20 mg/dL in vitro	20–500	—	Proof of concept
[32]	0.2–4	$S_{11},\;S_{21},\;S_{22},\;f$ SOLT Cal.	INNHO	training: 255×25 testing: 255×5 in vitro	20–500	$4.5\times 10^{-3}$	RMSE: 5.52 mg/dL *k*-fold cross-validation

**Table 7:** Performance comparison of previous publications regarding the iAUC120 value.

Reference	Value	Model	Performance	Diabetic Status
[114]	iAUC120	Boosted decision trees	R = 0.644	TGD
[115]	iAUC120	Boosted decision trees	R = 0.70	healthy
[116]	iAUC120	Boosted decision trees	R = 0.62	healthy

**Table 8:** Criteria for FDA and EMA according to [26,77].

Reference	Agency	Country	Blood Glucose Level	Min. Accuracy
[120]	EMA	EU	≥100 mg/dL	95%±15%
[119]	FDA	USA	≥75 mg/dL	95%±12%
				98%±15%

**Table 9:** Technical data comparison between FreeStyle Libre and Dexcom [22,23,80,123,126].

	FreeStyle Libre 2	FreeStyle Libre 3	Dexcom G6
**Release time**	2020	2021	2020
**Sensor type**	CGM using flash glucose monitoring system	CGM using CGM system	CGM
**Sensor principle**	electrochemical	electrochemical	electrochemical
**Regulatory** **status**	CE Mark cleared by FDA	CE Mark not cleared by FDA	CE Mark cleared by FDA
**Sensor size**	5 mm in height 35 mm in diameter	2.9 mm in height 21 mm in diameter	45.7 mm × 30.5 mm × 15.2 mm
**Sensor weight**	5 g	1 g	12 g
**BG measuring range**	40–500 mg/dL	40–500 mg/dL	40–400 mg/dL
**Working period**	14 days	14 days	10 days
**Calibration** **time**	60 min	60 min	120 min
**Wearing position**	back of the upper arm	back of the upper arm	belly (from the age of 2) back of the upper arm (from the age of 2) the upper buttocks (ages from 2 to 17)
**User age**	from the age of 4	from the age of 4	from the age of 2
**Data reading**	mobile phone (FreeStyle LibreLink APP) seperate reader	mobile phone	mobile phone (Dexcom Follow App)

**Table 10:** Review and comparison of different sensor systems with and without AI-based post-processing.

Reference	Evaluation Object	Measuring Method	Post- Processing	Detection Range (mg/dL)	Calibration/ Validation	Accuracy/ Sensitivity	Observation Time	Sensor Size	Influence Factor/ Sensor Limitation/ Further Development	Dataset
**Sensor Systems:**										
[29]	real saliva in vivo	electro- chemical	—	0–180	Proof of Concept	—	testing: 20 min; monitoring more than 5 h	25 mm × 5 mm × 0.5 mm	many proteins in the saliva	1 person
[137]	aqueous solution with, glucose, urea, lactate in vitro	Raman spectroscopy	filtering, smoothing, least-square fit	glucose: 18–1081 urea: 18–3604 lactate: 18–3604	area under Raman shift peaks	$R^{2}=0.97$ ≈4072 counts/mM	360 s each meas. 3×36×360 s	—	interference due to other blood comp., scattering light	3×36 meas.
[93]	real blood with NaCl, water and glucose in vitro	microwave	—	0–40.000 (14–16 GHz)	temperature control	reflected signal: 0.08${}^{\circ }$ and 3.2 mV (Δ10,000 mg/dL) transmitted signal: 0.2${}^{\circ }$ and 2 mV (Δ7500 mg/dL)	—	decimeter range plus VNA	temperature of the oscillator, sedimentation in the blood samples, water absorption	50×4 meas.
[98]	glucose water solution simulation	microwave	Debye model	0–500 (19 GHz)	—	phase of $S_{11}$ of 2° per 10 mg/dL	—	20×11.8×0.4 mm	tapering, fabrication errors	simulation
[99]	glucose solution in vitro	microwave	lin. regression	30–500 (1.5 GHz)	lin. regression	0.0049 dB/mg/dL	—	0.3×0.25 mm	optimization for more realistic situation	—
[104]	saline solutions in vitro	microwave	regression averaging	0–180 (1.45–1.55 GHz)	regression	21.7–23.4 dB/(mg/dL)	—	diameter: 25 mm thickness: 0.76 mm	optimization for mobility, data collecting time, data processing time	10×7 meas.
[138]	glucose water solution in vitro	microwave	lin. fitting	25–300 (0.8, 3.2 GHz)	lin. fitting, 2-port cal.	1.38 MHz per mg/dL	—	centimetre range plus VNA	temperature, geometrical parameters	12×3 meas.
[139]	glucose water solution in vitro	microwave	lin. fitting, averaging	0–400 (2.26 GHz)	VNA Cal.	1.947 mdB per mgdL${}^{-1}$µL	1080 s (CGM)	≈several centimetre plus VNA	temperature, rel. humidity	20×9 CGM meas.
[140]	real blood in vivo	microwave	lin. interpolation	89–262 (5.5, 8.5 GHz)	Comparison with Accu check and aqueous solution for cal. curve	8.5 GHz: 0.04 per mg/dL 5.5 GHz: 0.06 per mg/dL	—	30×18 mm plus VNA	temperature (skin, environ.), blood pressure, EMV, thickness of skin, pressure, sweat, pollution	11 persons
**Sensor Systems and Accuracy Improvement via Post-Processing:**										
[32]	glucose water solution in vitro	microwave	INNHO, LS-RANSAC, BPNN	20–500 (0.2–4 GHz)	Cal.: SOLT Val.: *k*-fold cross-val.	0.0045 dB/(mg/dL) RMSE of 5.52 mg/dL	—	80×30×6 mm	measurement uncertainty	training: 255×25 testing: 255×5
[35]	aqueous glucose water (in vitro) fingertip (in vivo)	microwave	PCA classification	40–140 (2.45 GHz)	VNA calibrated, internal validation	0.45–0.9 (dispersed) 0.63–1.25 (compact) each per MHz	1 h each 10 min	5.55×3 cm	temperature, geometrical parameters	600 samples (in vitro) 1 healthy P. (in vivo)
**Sensor Systems and Prediction of Blood Glucose Trends:**										
[83]	pig ears in vivo	Raman spectroscopy	Prediction MLR, PLSR	52–914	Lin. Regression for calibration, cross-4-fold validation	MARD: 6.6% R=0.96 (250–500 mg/dL) R=0.98 (>500 mg/dL)	3×7 h each 5 min	portable Raman spectrometer fibre bundle: 2 mm diameter	temperature, heart rate, skin movement, sweat, effective sampling volume	3 female Yorkshire pig
[141]	nail fold in vivo	Raman spectroscopy	Prediction PCA, BPNN	105–216	Cal. with 2 reference points	$R^{2}=0.98$ RMSE = 5 mg/dL	12×10×2.5 h each 5 min 6 meas.	Renishaw inVia confocal Raman spectrometer	temperature dirt, sweat	12 healthy persons
[87]	in vivo	impedance spectroscopy and multiple sensors	time series analysis sensor fusion	0–200 (1–150 kHz) (10–60 MHz)	Comparison with Accu-check and calibration	average correlation factor = 0.8314 NRMSE = 14.6064	3×72 h (diabetic, CGM) healthy: during lunch	flexible wrist band ≈ several cm	movement artifacts sweat	6 healthy, 3 diabetic persons
[97]	real blood in vivo	microwave	Prediction linear regression	60–400 (1.3 GHz)	Pre-processing Accu check as reference	MARD: 22.98% (without sub-band) 4.204% (with sub-band)	—	6.8×4.8 cm	object movement, temperature, pressure, humidity	75 non-diabetic 50 pre-diabetic 125 diabetic persons

**Table 11:** Performance comparison for 30 min prediction horizon.

Reference	Model	RMSE in mg/dL	Data Set
[117]	RNN	18.87	Ohio T1DM
[145]	RNN	19.04	Ohio T1DM
[146]	RNN	18.22	Ohio T1DM
[147]	Autoregression with exogenous inputs (ARX)	19.48	Ohio T1DM
[148]	Grammatical evolution (GE)	21.19	Ohio T1DM
[149]	Physiological models	19.33	Ohio T1DM
[150]	XGBoost	19.32	Ohio T1DM
[151]	Convolutional Neural Network (CNN)	21.72	Ohio T1DM
[42]	Ensemble MMS (3 aggregated NNs)	19.57	Ohio T1DM
[152]	Long short-term memory (LSTM)	18.23	Ohio T1DM
[153]	RNN and Restricted Boltzmann Machines (RNN-RBM)	15.59	DirecNet [154]
[155]	Support Vector Regression (SVR)	18.0	own data set
[156]	LSTM	21.4	described in [155]

## Abbreviations

The following abbreviations are used in this manuscript:

AA	Ascorbic Acid
AI	Artificial Intelligence
ARX	Autoregression with Exogenous Inputs
BG	Blood Glucose
BFGS Method	Broyden-Fletcher-Goldfarb-Shanno Method
BGL	Blood Glucose Level
BLE	Bluetooth Low Energy
BPNN	Back Propagation Neural Network
CA	Cellulose acetate
CGM	Continuous Glucose Monitoring
CCD	Charge-coupled Device
CEG	Clarke Error Grid
CNN	Convolutional Neural Network
CSRR	Complementary Split Ring Resonator
CVNN	Complex-Valued Neural Network
C6	Coumarin 6
D	Depth
DI	Deionized
DM	Diabetes Mellitus
EC	Ethyl Cellulose
EM	Electromagnetic
EMA	European Medicine Agency
FDA	Food and Drug Administration
FI	Fluorescence Intensities
FM	Frequency Number Matcher
GA	Glycidoxypropyltrimethoxysilane
GE	Grammatical Evolution
GOD	Glucose Oxidase
HIV	Human Immunodeficiency Virus
INNHO	Improved Neural Network and Hybrid Optimization
IPA	Isopropanol
ISM	Industrial, Scientific and Medical
LD	Laser Diode
LOD	Limit of Detection
LP	Lowpass Filter
LS-RANSAC	Least Squares-Random Sample Consensus
LSTM	Long short-term memory
MAE	Median Absolute Error
MARD	Mean Absolute Relative Difference
MG	Mouthguard
MLR	Multiple Linear Regression
MUT	Material Under Test
MSL	Microstrip Line
NA	Not a Number
NI	Non-invasive
NIR	Near Infrared
NRMSE	Normalized Root Mean Squared Error
OCT	Optical Coherence Tomography
OP	Optical Polarimetry
P	Persons
P-TiO${}_{2}$	Porous TiO${}_{2}$
PCA	Principal Component Analysis
PETG	Polyethylene Terephthalate Glycol
PBS	Phosphate Saline Buffer
PLSR	Partial Least Squares Regression
PNA	Power Network Analyser
PPG	Photoplethysmography
PPGR	Postprandial Glycemic Response
PtP	Platinum Meso-Tetra Porphyrin
PS	Polystyrene Particles
R	Regression
RBM	Restricted Boltzmann Machines
RF Regression	Random Forest Regression
RMSE	Root Mean Square Error
RNN	Recurrent Neural Network
RSD	Relative Standard Deviation
SEG	Surveillance Error Grid
SI	Ratio of FI and Glucose Concentration
SMBG	Self-Monitoring of Blood Glucose
SOLT	Short-Open-Load-Thru
SPP	Surface Plasmon Polariton
SVR	Support Vector Regression
T1D	Diabetes Mellitus Type 1
T2D	Diabetes Mellitus Type 2
TGD	Gestational Diabetes Mellitus
U	Unit
UA	Uric Acid
UWB	Ultra Wide Band
VNA	Vector Network Analyser
VOC	Volatile Organic Compound
WHO	World Health Organisation
